# A bacterial aromatic aldehyde dehydrogenase critical for the efficient catabolism of syringaldehyde

**DOI:** 10.1038/srep44422

**Published:** 2017-03-15

**Authors:** Naofumi Kamimura, Takayuki Goto, Kenji Takahashi, Daisuke Kasai, Yuichiro Otsuka, Masaya Nakamura, Yoshihiro Katayama, Masao Fukuda, Eiji Masai

**Affiliations:** 1 Department of Bioengineering, Nagaoka University of Technology, Nagaoka, Niigata 940-2188, Japan; 2 Forestry and Forest Products Research Institute, Tsukuba, Ibaraki 305-8687, Japan; 3 College of Bioresource Sciences, Nihon University, Fujisawa, Kanagawa 252-0880, Japan

## Abstract

Vanillin and syringaldehyde obtained from lignin are essential intermediates for the production of basic chemicals using microbial cell factories. However, in contrast to vanillin, the microbial conversion of syringaldehyde is poorly understood. Here, we identified an aromatic aldehyde dehydrogenase (ALDH) gene responsible for syringaldehyde catabolism from 20 putative ALDH genes of *Sphingobium* sp. strain SYK-6. All these genes were expressed in *Escherichia coli*, and nine gene products, including previously characterized BzaA, BzaB, and vanillin dehydrogenase (LigV), exhibited oxidation activities for syringaldehyde to produce syringate. Among these genes, SLG_28320 (*desV*) and *ligV* were most highly and constitutively transcribed in the SYK-6 cells. Disruption of *desV* in SYK-6 resulted in a significant reduction in growth on syringaldehyde and in syringaldehyde oxidation activity. Furthermore, a *desV ligV* double mutant almost completely lost its ability to grow on syringaldehyde. Purified DesV showed similar *k*_cat_*/K*_*m*_ values for syringaldehyde (2100 s^−1^·mM^−1^) and vanillin (1700 s^−1^·mM^−1^), whereas LigV substantially preferred vanillin (8800 s^−1^·mM^−1^) over syringaldehyde (1.4 s^−1^·mM^−1^). These results clearly demonstrate that *desV* plays a major role in syringaldehyde catabolism. Phylogenetic analyses showed that DesV-like ALDHs formed a distinct phylogenetic cluster separated from the vanillin dehydrogenase cluster.

Lignin is the second most abundant bio-resource on earth after cellulose, and its decomposition is essential for the carbon cycle. In nature, lignin is initially depolymerized via radical formation by oxidative enzymes that are secreted from ligninolytic fungi and bacteria^1^,^2^,^3^,^4^. The resultant pool of heterogeneous aromatic molecules with low molecular weights is predominantly assimilated by bacteria^5^,^6^. Most lignin-derived aromatics with guaiacyl (4-hydroxy-3-methoxyphenyl) and syringyl (4-hydroxy-3,5-dimethoxyphenyl) nuclei are catabolized through vanillin (VN) and syringaldehyde (SN), respectively, by a variety of enzymes in a specific manner; they are further degraded via the ring-cleavage pathways for protocatechuate, 3-*O*-methylgallate, and gallate^5^,^7^,^8^. Since VN and SN are readily obtained by chemical and thermal depolymerization of lignin^9^,^10^,^11^,^12^, they are useful intermediates, through microbial catabolism, in the production of industrially valued chemicals, such as 2-pyrone-4,6-dicarboxylate^13^,^14^,^15^, *cis, cis*-muconate^16^, and polyhydroxyalkanoate^17^ (Fig. 1). Therefore, understanding the genes and enzymes responsible for the conversion of VN and SN is essential for lignin valorization.

To date, VN dehydrogenase genes (*vdh*) have been studied extensively in various bacteria, particularly among Pseudomonads because *vdh* mutants produce VN, used for flavoring, from ferulate and eugenol^18^,^19^,^20^,^21^,^22^,^23^. The substrate range of some VN dehydrogenases for benzaldehyde derivatives, including SN, has been determined^24^,^25^,^26^; however, these aromatic dehydrogenases have weak SN oxidation activity. These findings imply the presence of other aromatic aldehyde dehydrogenase (ALDH) genes that are responsible for the oxidation of SN.

*Sphingobium* sp. SYK-6, an alphaproteobacterium, has the ability to grow on diverse lignin-derived biaryls and monoaryls^5^. Most of these lignin-derived biaryls with syringyl and guaiacyl moieties are degraded to syringate and vanillate via SN and VN, respectively. Over 30 genes of SYK-6 responsible for the upper funneling pathway and lower ring-cleavage pathways have been identified and characterized^5^. Among these genes, the VN dehydrogenase gene (*ligV*), which is essential for the catabolism of VN, was isolated by shotgun cloning^25^. The deduced amino acid sequence of *ligV* exhibited 35–53% identity with those of the known VN dehydrogenase genes of *Pseudomonas*^21^, *Rhodococcus*^27^, *Corynebacterium*^24^, and *Amycolatopsis*^18^. Even though LigV showed a broad range of activity against benzaldehyde derivatives, the activity for SN was considerably lower than that for VN. Further, disruption of *ligV* minimally affected the growth of SYK-6 on SN^25^. These results suggest that an alternative aromatic ALDH gene is involved in the catabolism of SN in SYK-6. Previously, we tried to isolate the SYK-6 SN dehydrogenase gene using the same procedure employed for the cloning of *ligV*. Consequently, *bzaA* and *bzaB*, whose products were capable of converting a wide range of benzaldehyde derivatives including SN, were isolated^28^. However, disruption of these genes in SYK-6 had only a slight impact on its growth on SN^28^.

In this study, in order to identify the SN dehydrogenase gene involved in SN catabolism in SYK-6, we carried out a genome-wide screening for the gene. Based on the SN oxidation activities of the products of 20 putative ALDH genes and their expression levels in the SYK-6 cells, a reduced number of candidate genes were examined further. Gene disruption experiments identified the actual SN dehydrogenase gene, and enzymatic properties of SN dehydrogenase and LigV were characterized.

## Results and Discussion

### Characterization of SN conversion by cell extracts of *Sphingobium* sp. SYK-6

To obtain information on properties of the enzymes involved in the conversion of SN in SYK-6, coenzyme requirements and induction profiles of the enzyme activity were examined. When an extract of SYK-6 cells grown in LB (300 μg of protein/ml) was incubated with 300 μM SN in the presence of 500 μM NAD^+^, the extract converted SN into syringate with a specific activity of 34 ± 0.3 nmol·min^−1^·mg^−1^. This activity was 7.0- and 14-fold higher than those obtained in the presence of NADP^+^ and in the absence of a coenzyme, respectively. These results indicated that NAD^+^-dependent aromatic ALDHs are involved in the oxidation of SN in SYK-6.

Our previous work suggested that SN conversion was constitutive in SYK-6 when LB was used as a non-inducing condition^25^. To confirm the inducibility of SN conversion, enzyme activities of extracts of SYK-6 cells grown in a minimal medium, Wx-SEMP, supplemented with or without 5 mM SN or VN, were evaluated. In the presence of NAD^+^, the SN oxidation activities of the extracts from cells grown in Wx-SEMP (32 ± 1.5 nmol·min^−1^·mg^−1^) and Wx-SEMP with VN (31 ± 2.9 nmol·min^−1^·mg^−1^) were almost identical to that of cells grown with SN (30 ± 2.2 nmol·min^−1^·mg^−1^). These results suggest that the genes responsible for the oxidation of SN are constitutively expressed.

### Genome-wide screening of candidates for the SN dehydrogenase gene

**Figure 2:**
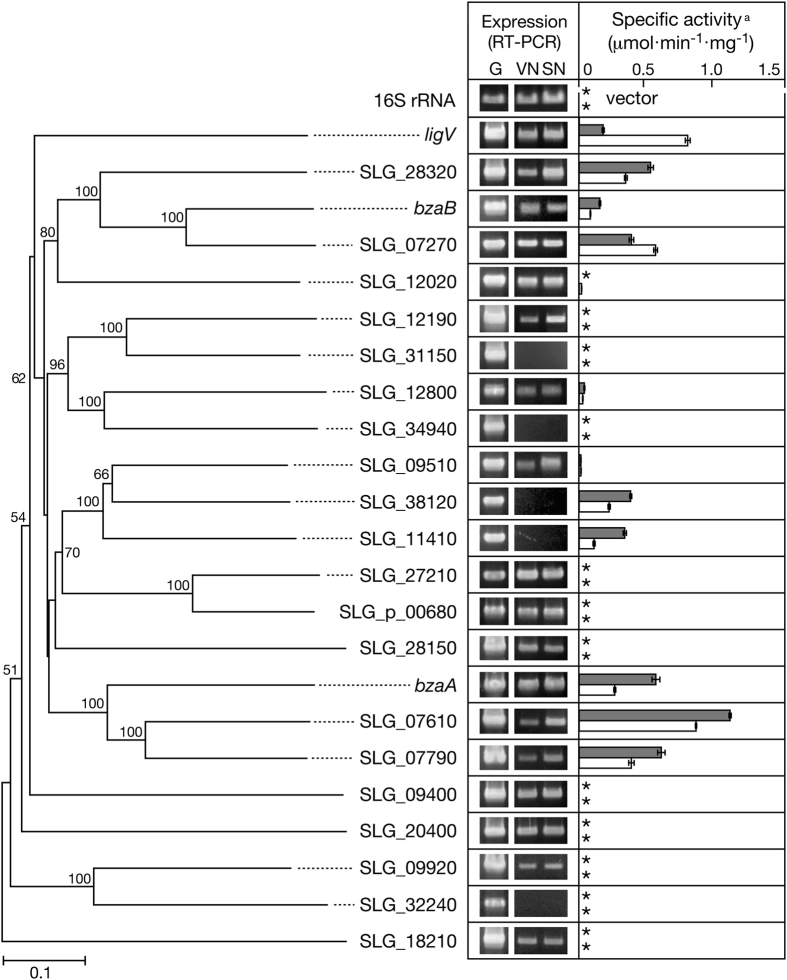
Genome mining for SN dehydrogenase genes in *Sphingobium* sp. SYK-6. (Left) Phylogenetic tree based on the alignment of the deduced amino acid sequences of *ligV, bzaA, bzaB*, and 20 putative ALDH genes in SYK-6. The scale bar corresponds to 0.1 amino acid substitutions per position. Bootstrap values (based on 1000 replicates) are given at branch points. (Center) RT-PCR analyses of expression of the ALDH genes. Total RNA of cells grown in Wx-SEMP containing 5 mM of VN (VN) or SN (SN) was used as templates. Lane G indicates positive controls using the genomic DNA of SYK-6. (Right) Oxidation activities for SN (gray) and VN (white) of cell extracts of *E. coli* carrying each ALDH gene. The data are mean ± standard deviation of three independent experiments. ^a^Specific activities were normalized to their expression levels calculated from a gel image of SDS-PAGE (Table S2). Asterisks indicate activities lower than 0.01 μmol·min^−1^·mg^−1^.

The genome sequence of SYK-6 revealed the presence of 20 putative ALDH genes in addition to the previously characterized *ligV, bzaA*, and *bzaB* (Table S1). Phylogenetic analysis of 23 ALDHs in SYK-6 revealed that *ligV, bzaA*, and *bzaB* are placed into different clades (Fig. 2). *bzaA* clusters with SLG_07610 and SLG_07790 sharing 46 to 57% amino acid sequence identity, while *bzaB* clusters with SLG_07270 and SLG_28320 sharing 46 to 63% identity. To reduce the number of candidate genes, the presence of transcripts of all ALDH genes in SYK-6 were evaluated by reverse transcription (RT)-PCR analyses using total RNA prepared from SYK-6 cells grown with SN or VN. DNA fragments with expected sizes were amplified for 18 ALDH genes (Fig. 2). In contrast, no amplification products of SLG_31150, SLG_34940, SLG_38120, SLG_11410, and SLG_32240 were obtained from RNA isolated from cells grown in either culture condition (Fig. 2).

To investigate the ability of putative ALDH gene products to oxidize SN, all ALDH genes were PCR amplified and cloned into pET21a(+) and expressed in *E. coli* cells harboring the resultant plasmids. SDS-polyacrylamide gel electrophoresis (SDS-PAGE) analysis of cell extracts of the *E. coli* transformants showed successful expression of all the genes except SLG_32240 and SLG_34940 (Fig. S1). Cell extracts of the *E. coli* transformants (10–100 μg of protein/ml) were incubated with 100 μM SN or VN in the presence of 500 μM NAD^+^ to evaluate their SN and VN oxidation activities (Fig. 2 and Fig. S2). To account for the different levels of expression of each ALDH gene, specific activities were normalized to their expression levels calculated from a gel image of SDS-PAGE (Table S2). As a result, eight gene products, including the products of SLG_28320, *bzaB*, SLG_07270, SLG_38120, SLG_11410, *bzaA*, SLG_07610, and SLG_07790, had specific activities toward SN that were the same as or higher than the gene product of *ligV* (80–620%). All these aromatic ALDH gene products converted SN into syringate (Fig. S2). Notably, the gene product of SLG_07610 had the highest SN dehydrogenase activity (1.11 ± 0.01 μmol·min^−1^·mg^−1^). Most gene products showed lower oxidation activities toward VN (33–72%); however, the gene products of *ligV* and SLG_07270 had higher activity toward VN than to SN.

Based on the transcriptional level of ALDH genes in SYK-6 cells and the SN oxidation activities of the gene products, four genes, SLG_28320, SLG_07270, SLG_07610, and SLG_07790, were selected as candidates for SN dehydrogenase genes involved in SN catabolism in SYK-6 and were further characterized.

### Substrate range of the putative SN dehydrogenases

In order to characterize the substrate range of the candidates for SN dehydrogenase, the oxidation activities of cell extracts of *E. coli* carrying SLG_07270, SLG_07610, SLG_07790, and SLG_28320 along with *ligV, bzaA*, and *bzaB* were examined using a spectrophotometric lactate dehydrogenase-coupled assay with various aromatic aldehydes including SN, VN, benzaldehyde, *p*-hydroxybenzaldehyde, protocatechualdehyde, *m*-anisaldehyde, veratraldehyde, coniferyl aldehyde, salicylaldehyde, and *m*-hydroxybenzaldehyde (Fig. S3). The gene products of *bzaA*, SLG_07790, and SLG_28320 exhibited oxidation activities toward all of the substrates (Table 1). The highest activities of LigV, BzaA, SLG_07610, and SLG_07790 were observed when *m*-hydroxybenzaldehyde was used as a substrate. In contrast, BzaB and SLG_07270 shared similar substrate ranges and showed the highest activities toward *m*-anisaldehyde. These two enzymes share relatively high amino acid sequence similarities (63% identity), implying a correlation between their substrate specificity and their amino acid sequence similarity.

### Transcriptional levels of the putative SN dehydrogenase genes in SYK-6

**Figure 3:**
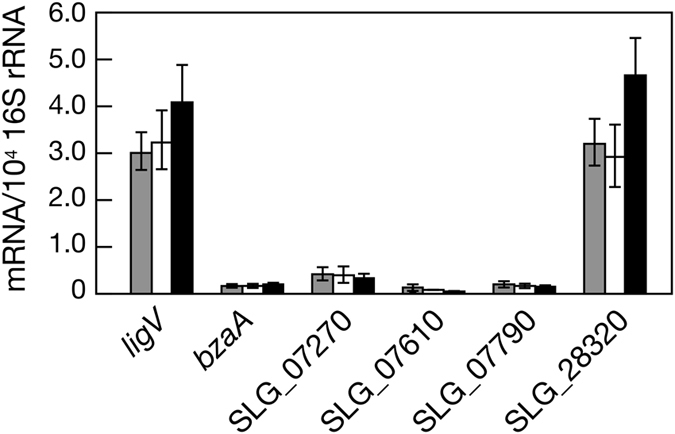
qRT-PCR analysis of the expression of ALDH genes in *Sphingobium* sp. SYK-6. Relative mRNA abundance was determined for *ligV, bzaA*, SLG_07270, SLG_7610, SLG_07790, and SLG_28320. Total RNA was isolated from SYK-6 cells grown in Wx-SEMP containing 5 mM SN (gray), Wx-SEMP containing 5 mM VN (white), and Wx-SEMP (black). Values for each mRNA level were normalized to 16S rRNA. The data are mean ± standard deviation of three independent experiments.

Because the gene(s) responsible for the oxidation of SN may be constitutively expressed in SYK-6, we predicted that genes that are highly transcribed must be involved in SN catabolism. To accurately determine the transcriptional levels of the candidate genes, qRT-PCR analyses for *ligV, bzaA*, SLG_07270, SLG_07610, SLG_07790, and SLG_28320 were performed using the total RNA isolated from SYK-6 cells grown with or without 5 mM of SN or VN. All six genes showed constitutive transcription, and *ligV* and SLG_28320 were transcribed at similar and much higher levels than other genes (Fig. 3). In contrast, mRNA levels of *bzaA*, SLG_07270, SLG_07610, and SLG_07790 in cells grown in SEMP were only 1.5 to 9.6% of that of *ligV* (Fig. 3). These results may suggest that SLG_28320 and *ligV* play a major role in the catabolism of SN.

### Role of SLG_28320 and *ligV* in the catabolism of SN and VN

**Figure 4:**
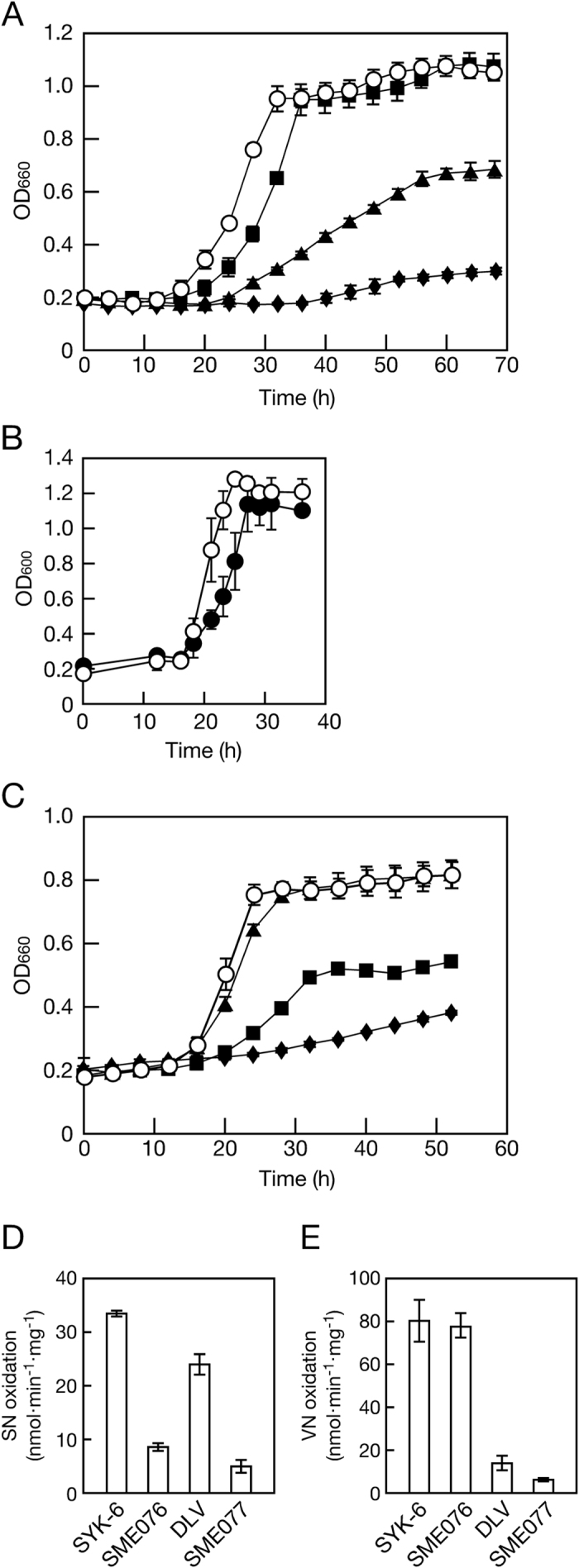
Characterization of the SLG_28320 and *ligV* knockout mutants. (**A** and **C**) Growth of SYK-6 (white circles), SLG_28320 mutant (SME076, black triangles), *ligV* mutant (DLV, black squares), and SLG_28320 *ligV* double mutant (SME077, black diamonds) on Wx medium containing 5 mM SN (**A**) and VN (**C**). Cell growth was measured by optical density at 660 nm. (**B**) Growth of SME076 harboring pJB28320 on SN. SYK-6 cells harboring pJB866 (white circles) and SME076 cells harboring pJB28320 (black circles) were incubated in Wx medium containing 5 mM SN. Cell growth was measured by optical density at 600 nm. (**D** and **E**) Oxidation activities of cell extracts of SYK-6, SME076, DLV, and SME077 toward SN (**D**) and VN (**E**). The data are mean ± standard deviation of three independent experiments.

To examine whether SLG_28320 and *ligV* are involved in the catabolism of SN, gene knockout mutants were created by the insertion of chloramphenicol or kanamycin resistance genes. These mutations were confirmed by Southern hybridization analyses (Fig. S4). The growth of SLG_28320 mutant (SME076), *ligV* mutant (DLV)^25^, and SLG_28320 *ligV* double mutant (SME 077) on 5 mM SN was compared to that of SYK-6 (Fig. 4A). The growth rate of SME076 on SN was significantly reduced, and the final biomass yield was almost 65% that of the wild type. To determine if this growth defect was caused by the disruption of SLG_28320, pJB866^29^ carrying SLG_28320 (pJB28320) was introduced into SME076 cells. The SME076 cells harboring pJB28320 grew on SN as well as the wild type (Fig. 4B). These results indicate that SLG_28320 is indeed involved in SN catabolism in SYK-6; therefore, this gene was designated *desV*. DLV showed a moderate growth reduction on SN as reported in our previous study^25^; however, SME077 almost completely lost the ability to grow on SN (Fig. 4A). These results indicate that both *desV* and *ligV* are necessary for the efficient conversion of SN, and *desV* plays a dominant role in SN transformation. We also examined the involvement of *desV* in growth on VN (Fig. 4C). While a significant reduction in growth was observed in DLV, SME076 showed almost the same growth as the wild type. However, further growth reductions were observed in SME077, suggesting that *desV* also contributes, in part, to VN catabolism.

To further investigate the level of involvement of *desV* and *ligV* in the catabolism of SN and VN, the oxidation activities for SN and VN of SME076, DLV, and SME077 were determined using cell extracts (300 μg of protein/ml) (Fig. 4D and E). Whereas the SN oxidation activity of SME076 was significantly decreased (26% that of the wild type), DLV retained 72% of the original activity. SME077 showed only 15% of the wild-type activity. The drastic decrease in SN oxidation activity in SME076 caused by the disruption of *desV* appears to result in the slow growth of SME076 cells on SN and their low final biomass yield. In contrast, no substantial loss of VN oxidation activity was observed in SME076. However, SME077 exhibited a further decline in activity when compared to that of DLV. These results correspond with those obtained in the growth assays.

### Purification of DesV and LigV

The coding regions of *desV* and *ligV* were each cloned into pET-16b, and His-tag fused *desV* and *ligV* were expressed in *E. coli* BL21(DE3). SDS-PAGE analyses revealed the production of proteins 57 kDa and 51 kDa in size (Fig. S5A), which are similar to the values calculated from the deduced amino acid sequences of His-tag fused *desV (M*_*r*_. 55,173) and *ligV (M*_*r*_. 52,879), respectively. DesV and LigV were purified to near homogeneity by Ni affinity chromatography. Using size exclusion chromatography, the native molecular masses of purified DesV and LigV were estimated to be 120 kDa and 220 kDa, respectively (Fig. S5B). Furthermore, similar results were obtained by native-PAGE (Fig. S5C). Based on the size of the monomers, DesV and LigV were deduced to be a dimer and a tetramer, respectively. Previously, a dimer and a tetramer of VN dehydrogenases have been reported from *Burkholderia cepacia* TM1 and *Micrococcus* sp. TA1, respectively^26^.

### Enzymatic properties of DesV and LigV

Purified DesV and LigV showed the same optimal temperature (50 °C) and optimal pH (10.0) (Fig. S6). A similar optimal temperature and pH were reported from VN dehydrogenases of *B. cepacia* TM1 and *Micrococcus* sp. TA1^26^.

DesV could oxidize SN in the presence of either NAD^+^ or NADP^+^; however, the specific activity was 7.3-fold higher when NAD^+^ was used (0.99 μmol·min^−1^ mg^−1^). In contrast, LigV specifically required NAD^+^ (0.33 μmol·min^−1^·mg^−1^ with NAD^+^ and no activity with NADP^+^). The UV-visible spectra of both DesV and LigV showed no absorbance related to bound flavin cofactors (data not shown).

The kinetic parameters of the purified DesV and LigV for SN and VN were determined (Table 2 and Fig. S7). These kinetic data clearly demonstrate that LigV functions as a VN dehydrogenase, while DesV has the potential to function as a dehydrogenase for both SN and VN. To date, kinetic parameters of aromatic ALDHs toward SN have not been reported, while the parameters toward VN were determined for VN dehydrogenases from *Micrococcus* sp. TA1^26^, *B. cepacia* TM1^26^, and *Corynebacterium glutamicum* ATCC 13032^24^, *p*-hydroxy benzaldehyde dehydrogenase from *Acinetobacter* sp. ADP1^30^, and ALDH1A1 and ALDH3A1 from humans^31^ (Table S3). LigV and DesV exhibited the lowest *K*_*m*_ and the highest *k*_cat_/*K*_*m*_ values (Table S3). These results raise the question of why the SN oxidation activity of DLV was significantly reduced despite LigV exhibiting significantly low catalytic efficiency toward SN (Fig. 4 and Table 2). LigV may have a role in the conversion of SN when its cellular concentration is high because *ligV* and *desV* showed similar expression levels in the SYK-6 cells, and little difference exists between the *k*_cat_ values of DesV and LigV for SN (0.99 s^−1^ and 0.59 s^−1^), although the *K*_*m*_ value of LigV for SN is very high (412 μM). In contrast, a double mutant of *desV* and *ligV* (SME077) still retained a poor ability to grow on and convert SN and VN (Fig. 4). Moreover, the specific activities for the conversion of SN (5.2 nmol·min^−1^·mg^−1^) and VN (7.1 nmol·min^−1^·mg^−1^) with NAD^+^ in SME077 were significantly higher than those of the wild type in the absence of NAD^+^ (SN, 2.3 nmol·min^−1^·mg^−1^; VN, 0.8 nmol·min^−1^·mg^−1^). These results suggest that the remaining activities for the conversion of SN and VN in SME077 were derived from other NAD^+^-dependent aromatic ALDHs.

Involvement of multiple ALDH genes for the conversion of VN was also shown in *Pseudomonas putida* KT2440^32^. Although *P. putida* KT2440 has a *vdh* gene (PP_3357), disruption of this gene does not affect growth on VN. In contrast, proteomic analyses indicated that the production of other ALDHs, PP_0545, PP_1948, PP_2680, PP_3151, PP_5120, and PP_5258, increased in response to VN. Additional disruption of PP_0545, PP_1948, and PP_2680 in *vdh* mutant enhanced productivity of VN from ferulate^23^. Another example of the involvement of multiple ALDH genes in the assimilation of VN was reported in *C. glutamicum* ATCC 13032^24^. In this strain, *vdh* plays an important role in the degradation of VN. However, catabolism of VN was still observed in a *vdh* mutant, suggesting the presence of alternative ALDH genes for the oxidation of VN. In addition, the disruption of *vdh* showed no effect on growth on SN. In the genome sequence of *C. glutamicum* ATCC 13032 (NC_003450), we found seven putative ALDH genes in addition to *vdh*. Subsequent phylogenetic analysis indicated the presence of an ALDH gene (NCgl0523) whose deduced amino acid sequence shared 38% identity with that of *desV* (Fig. S8). NCgl0523 may be involved in the conversion of VN and SN in *C. glutamicum* ATCC 13032.

### Phylogenetic analyses of aromatic ALDH genes

**Figure 5:**
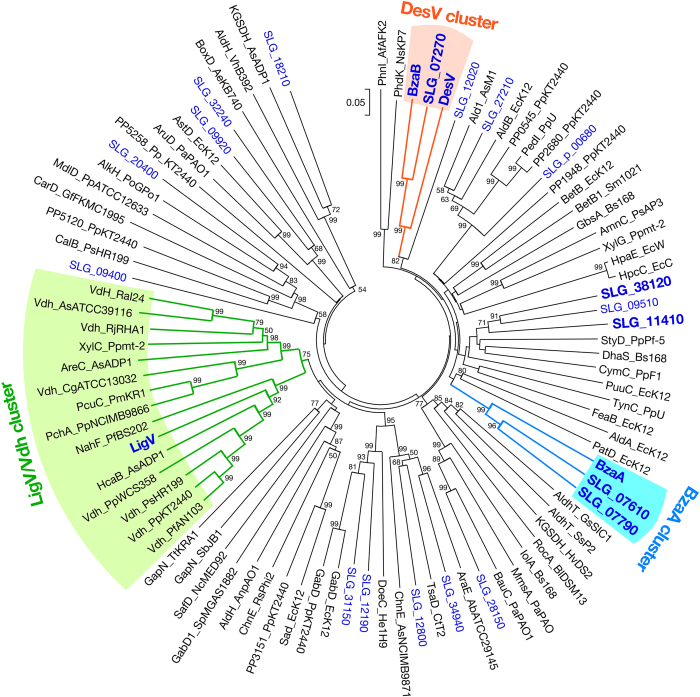
Phylogenetic tree of ALDHs from SYK-6 and other bacterial ALDHs. The tree was constructed using neighbor-joining with 1000 bootstrap replicates. Bootstrap values are indicated at the nodes, and the scale corresponds to 0.05 amino acid substitutions per position. Accession numbers and the species are given in Table S4. ALDHs from SYK-6 are shown in blue, and ALDHs which had SN oxidation activity are indicated in bold blue.

To gain insight into the evolutionary relationship between the SYK-6 aromatic ALDH genes, whose products exhibited oxidation activities for VN and SN, and other aromatic ALDH genes, a phylogenetic analysis of the SYK-6 ALDH genes was conducted with known bacterial ALDH genes (Fig. 5 and Table S4). Nine SYK-6 aromatic ALDH genes, the products of which showed SN oxidation activities, are divided into four clusters. SLG_11410 and SLG_38120 clustered with phenylacetaldehyde dehydrogenase (StyD)^33^, *p*-cumic aldehyde dehydrogenase (CymC)^34^, and 3-hydroxypropionaldehyde dehydrogenase (DhaS)^35^. LigV was closely related to Vdhs of *Pseudomonas* strains^23^,^36^,^37^,^38^, salicylaldehyde dehydrogenase (NahF)^39^, and hydroxybenzaldehyde dehydrogenase (HcaB)^30^. Although Vdhs from Gram-positive bacteria, including *Rhodococcus*^27^, *Corynebacterium*^24^, and *Amycolatopsis*^18^, are minimally distinguished from LigV and Pseudomonad Vdhs, they form a single cluster (the LigV/Vdh cluster). In contrast, DesV–BzaB–SLG_07270 and BzaA–SLG_07610–SLG_07790 are distinct from known ALDHs and form independent clusters designated the DesV cluster and BzaA cluster, respectively. A conserved domain search revealed that DesV–BzaB–SLG_07270, BzaA–SLG_07610–SLG_07790, and LigV/Vdh contain ALDH_AldA-Rv0768 (cd07139), ALDH_AldA-AAD23400 (cd07106), and ALDH_SaliADH (cd07105) motifs, respectively. In all these enzymes, essential catalytic amino acid residues Cys297, Glu263, Gly294, and Asn166 (DesV numbering) are completely conserved^40^. In addition, the Gly241 and Gly246 (DesV numbering) essential residues of the ALDH Rossmann fold necessary for cofactor binding are also conserved^40^.

Aromatic ALDH genes affiliated with the DesV cluster, BzaA cluster, and LigV/Vdh cluster in living organisms were surveyed using the co-occurrence tool of the STRING database (Fig. S9). The genes which showed the highest amino acid sequence identities with DesV (31–65%), BzaA (39–64%), and LigV (29–84%) were found from in 37 taxa of bacteria, eukaryotes, and archaea (Table S5). The phylogenetic tree constructed using the amino acid sequences of the above genes showed that almost half of the extracted genes belonged to one of the three clusters (Fig. S10). When compared to the DesV and BzaA types of aromatic ALDH genes, the LigV/Vdh-type aromatic ALDH genes are more broadly distributed among bacteria. The aromatic ALDH genes in the DesV and BzaA clusters were found not only in the SYK-6-related Sphingomonad strains but also in other Proteobacteria and Actinobacteria. These observations may imply that, in addition to the LigV/Vdh cluster genes, aromatic ALDH genes classified into the DesV and BzaA clusters also generally participate in the catabolism of lignin-derived aromatic compounds. For example, *Xanthomonas oryzae*^41^, a gammaproteobacterial pathogen of rice, has a DesV ortholog, XOC_0933 which exhibited 65% amino acid sequence identity with DesV (Fig. S10). Interestingly, its proximal gene, XOC_0934, showed 61% amino acid sequence identity with the feruloyl-coenzyme A (CoA) hydratase/lyase gene (*ferB*) of SYK-6 that is responsible for the conversion of feruloyl-CoA to VN^42^. In addition, the gene set of XOC_0933–XOC_0934 was also found in several members of *Xanthomonas* including *Xanthomonas axonopodis, Xanthomonas campestris*, and *Xanthomonas citri*. These findings may suggest that the DesV-type aromatic ALDH gene is involved in the catabolism of plant-derived aromatic compounds such as ferulate in *Xanthomonas*.

## Conclusions

The goal of this study was to identify the aromatic ALDH gene responsible for the conversion of SN from 20 putative ALDH genes in *Sphingobium* sp. SYK-6. For the first time, we were able to successfully identify *desV* as the SN dehydrogenase gene mainly involved in SN catabolism. Detailed information on a number of aromatic ALDH genes obtained in this study will be useful for application to biological lignin valorization. To establish economically viable biofuel production, increasing the value of lignin is essential. Recently, some approaches combining the chemical deconstruction of lignin and the microbial conversion of the resultant heterologous aromatic compounds have been attempted^17^,^43^. Since VN and SN are generally major intermediates of both microbial catabolism and chemical decomposition, aromatic ALDHs with a high catalytic efficiency toward these aromatic aldehydes, such as LigV and DesV, are extremely valuable for the production of chemicals from lignin.

## Methods

### Bacterial strains, plasmids, and culture conditions

The bacterial strains and plasmids used in this study are listed in Table S6. *Sphingobium* sp. SYK-6 and its mutant derivatives were routinely grown at 30 °C in Lysogeny broth (LB) or Wx minimal salt medium^44^ containing 5 mM SN, 5 mM VN, or SEMP (10 mM sucrose, 10 mM glutamate, 0.13 mM methionine, and 10 mM proline). When necessary, 50 mg of kanamycin (Km)/liter, 30 mg of chloramphenicol (Cm)/liter, and 12.5 mg of tetracycline (Tc)/liter were added to the cultures. *E. coli* strains were grown in LB at 37 °C. For cultures of cells carrying antibiotic resistance markers, the media for *E. coli* transformants were supplemented with 100 mg of ampicillin/liter, 25 mg of Km/liter, and 12.5 mg of Tc/liter.

### Enzyme assays using cell extracts of SYK-6 and its mutants

Cells of SYK-6 and its mutants (DLV, SME076, and SME077) grown in LB were inoculated into the same medium (final concentration, 1%) and further incubated for 12 h. The resultant cells were washed twice with 100 mM KH_2_PO_4_-K_2_HPO_4_ buffer (pH 7.0, buffer A). Cells resuspended in the same buffer were then broken by an ultrasonic disintegrator (UD-201; Tomy Seiko Co.). After centrifugation (19,000× g for 15 min at 4 °C), the supernatants were obtained as cell extracts. The protein concentration was determined by the Bradford method with bovine serum albumin as the standard. Cell extracts (300 μg of protein/ml) were incubated with 300 μM SN or 300 μM VN in the presence and absence of 500 μM of NAD^+^ or NADP^+^ at 30 °C. After incubation for 0.5 and 5.0 min, portions of the mixture were collected, and reactions were terminated by mixing them with the same volume of 0.2 N HCl. Supernatants obtained by centrifugation (19,000×g for 15 min at 4 °C) were filtrated and analyzed by high-performance liquid chromatography (HPLC; Acquity UPLC system; Waters) using a TSKgel ODS-140HTP column (2.1 by 100 mm; Tosoh) as described previously^45^. The mobile phase of the HPLC system was a mixture of water (75%) and acetonitrile (25%) containing formic acid (0.1%) at a flow rate of 0.3 ml/min. SN and VN were detected at 279 and 308 nm, respectively. The specific activity was expressed in moles of SN and VN converted per min per milligram of protein. For the determination of cofactor requirements, a centrifugal filtration of cell extracts was carried out with Amicon Ultra 3k (Millipore) to remove cofactors, and then the resultant fractions were used for the enzyme reaction. To examine the induction profile, cells of SYK-6 grown in LB were washed with Wx medium and resuspended in Wx-SEMP medium to an optical density at 600 nm (OD_600_) of 0.2. Once cultures reached an OD_600_ of 0.5 to 0.6, 5 mM SN or 5 mM VN was added to the cultures. After 6 h of further incubation, cell extracts were prepared and used for the enzyme assay.

### Genome search of putative ALDH genes

For the first step to search ALDH genes in the SYK-6 genome (AP012222 and AP012223), protein BLAST (BLASTP) was carried out using the deduced amino acid sequences of *ligV, bzaA*, and *bzaB* in the NCBI database. The BLASTP searches were then repeated using the homologous sequences found above to obtain more candidate genes. Pairwise alignments were performed using the EMBOSS Needle program through EMBL-EBI server (http://www.ebi.ac.uk/services)^46^. For phylogenetic analysis, multiple alignments were performed using the Clustal W program in MEGA software^47^, and then phylogenetic trees were constructed using the neighbor-joining algorithm of MEGA 7, employing 1000 bootstrap replicates.

### RT-PCR and qRT-PCR analysis

After 6 h of incubation of SYK-6 cells in the presence and absence of 5 mM SN or VN, 2 ml of cultures were harvested. Total RNA was isolated using Isogen II (Nippon Gene Co., Ltd.), followed by treatment with RNase-free DNase I (Roche). PrimeScript reverse transcriptase (Takara Bio Inc.) was used to synthesize cDNA from 2 μg of total RNA with random hexamer primer. A control PCR was performed with reverse transcriptase-negative samples to verify the absence of genomic DNA contamination. RT-PCR was performed with the resultant cDNA, specific primers, and Ex*Taq* DNA polymerase (Takara Bio Inc.). qRT-PCR was carried out with a Fast SYBR green master mix (Applied Biosystems) and StepOne Real-time PCR System (Applied Biosystems). To normalize the amount of RNA in each sample, 16S rRNA was used as an internal standard. The primers used for the analyses are listed in Table S7.

### Expression of SYK-6 ALDH genes

*bzaA, bzaB*, and other putative ALDH genes were amplified by PCR using PrimeSTAR GXL DNA Polymerase (Takara Bio Inc.) and primer sets listed in Table S7. The amplified fragments were cloned into pBluescript II KS(+) or pT7Blue, and then the nucleotide sequences were determined. The 1.4 to 1.5-kb NdeI-BamHI or NdeI-XhoI fragments from the resulting plasmids were cloned into the corresponding sites of pET21a(+) to yield the expression plasmids. *E. coli* BL21(DE3) cells harboring each expression plasmid were grown in LB at 30 °C, and the expression of the genes was induced for 4 h at 30 °C by adding 1 mM isopropyl-β-D-thiogalactopyranoside (IPTG) when the OD_600_ of the culture reached 0.5. The resultant cultures were washed twice with buffer A, and then the cells resuspended in the same buffer were broken by an ultrasonic disintegrator. After centrifugation (19,000× g for 15 min at 4 °C), the supernatants were obtained as cell extracts. The expression of the genes was confirmed using SDS-12% PAGE. Cell extracts of the *E. coli* transformants (10–100 μg of protein/ml) were incubated with 100 μM SN or VN in the presence of 500 μM NAD^+^ at 30 °C. After incubation for 0.5 and 5.0 min, portions of the mixture were collected and the amounts of substrates were measured using HPLC. For the calculation of the specific activities of the ALDHs for the oxidations of SN and VN, the signal intensities of each band of the ALDH in SDS-PAGE were quantified using a LumiVision image analyzer (Aisin Seiki Co., Ltd). Specific activities were expressed in moles of SN and VN converted per min per milligram of protein, which was normalized to the expression level of LigV.

### Substrate range

Enzyme assays to examine the substrate range of the gene products of *ligV, bzaA, bzaB*, SLG_07270, SLG_07610, SLG_07790, and SLG_28320 (*desV*) were performed according to the method described previously^25^,^37^. Cell extracts of the *E. coli* transformants (100–500 μg of protein/ml) were incubated with 100 μM substrate (Fig. S3), 500 μM NAD^+^, 1.2 mM pyruvate, 1.0 U lactate dehydrogenase in buffer A at 30 °C. The decrease in the amount of substrates was measured spectrophotometrically using a spectrophotometer (DU-7500, Beckman-Coulter). Specific activities were expressed in moles of substrates converted per min per milligram of protein. The concentrations of each substrate were calculated with the following extinction coefficient values: SN (ε_364_ = 8,936 M^−1^·cm^−1^), VN (ε_346_ = 8,697 M^−1^·cm^−1^), benzaldehyde (ε_293_ = 1,270 M^−1^·cm^−1^), *p*-hydroxybenzaldehyde (ε_331_ = 7,088 M^−1^·cm^−1^), protocatechualdehyde (ε_341_ = 9,070 M^−1^·cm^−1^), *m*-anisaldehyde (ε_330_ = 2,181 M^−1^·cm^−1^), veratraldehyde (ε_330_ = 3,536 M^−1^·cm^−1^), coniferyl aldehyde (ε_410_ = 3,330 M^−1^·cm^−1^), salicylaldehyde (ε_340_ = 2,656 M^−1^·cm^−1^), and *m*-hydroxybenzaldehyde (ε_318_ = 2,985 M^−1^·cm^−1^).

### Construction of mutants

For the construction of a *desV*-disruption plasmid, a 1.5-kb HindIII-XbaI fragment carrying *desV* from pKS2832 was cloned into the same sites of pK18*mobsacB* with the insertion of a Cm resistance gene. The resulting plasmids, pK18-2832Cm was introduced into SYK-6 cells by electroporation, and the candidates for *desV* mutant (SME076) were isolated as described previously^48^. Similarly, the candidates for *desV ligV* double mutant (SME077) were obtained by introduction of a *ligV*-disruption plasmid, pIK34D^25^ into SME076 cells. The disruption of each gene was confirmed by Southern hybridization analysis using digoxigenin system (Roche). The growth of the resulting mutant cells in Wx medium containing 5 mM SN or VN were examined by monitoring of OD_660_ automatically every 4 h using Bio-Photorecorder (TVS062CA, Advantec). A complementary plasmid, pJB28320 was constructed by cloning a DNA fragment carrying *desV* into pJB866^29^. The resulting plasmid was introduced into the cells of SME076 by electroporation, and the growth of the transformant in Wx medium containing 5 mM SN was examined.

### Purification of DesV and LigV

The 1.5-kb NdeI-BamHI fragment carrying *desV* and the 1.9-kb NdeI-XhoI fragment carrying *ligV* from pT21-2832 and pLVH, respectively, were ligated into the corresponding sites of pET-16b. The resultant plasmids, pT16-desV and pT16-ligV, were independently introduced into *E. coli* BL21(DE3), and then the His tag-fused *desV* and *ligV* were expressed. For purification, cell extracts were applied to His GraviTrap TALON columns (GE Healthcare). Purified fractions were subjected to desalting and centrifugal filtration with Amicon Ultra 30k (Millipore), and stored at −30 °C until use. The purity of the preparations was examined by SDS–12% PAGE.

### Determination of molecular mass

Purified DesV (100 μg/26 μl) and LigV (100 μg/32 μl) were subjected to size exclusion chromatography on a Superdex200 10/300GL column (GE Healthcare) eluted with 50 mM KH_2_PO_4_-K_2_HPO_4_ buffer (pH 7.0) containing 150 mM NaCl at a flow rate of 0.5 ml/min as described previously^45^. Native PAGE was performed using a 5–20% polyacrylamide gradient gel with a high-molecular-weight calibration kit for native electrophoresis (GE Healthcare).

### Enzyme characterization

The enzyme reaction was typically carried out in a 100 μl reaction mixture containing buffer A, DesV (20 μg/ml of protein, 180 nM dimer) or LigV (20 μg/ml of protein, 95 nM tetramer), and 500 μM NAD^+^ or NADP^+^. After the incubation (25 sec for the kinetic analysis and 60 sec for the determination of optimal pH and temperature, and coenzyme requirement), the reaction was terminated by the addition of the same volume of 0.2 N HCl. The amounts of the substrates and products were measured using HPLC. Specific activities were expressed in moles of syringate or vanillate produced per min per milligram of protein at 30 °C. The optimal temperatures were determined using buffer A (15 to 70 °C), and the optimal pH were examined using 50 mM GTA buffer (50 mM 3,3-dimethylglutarate, 50 mM Tris, and 50 mM 2-amino-2-methyl-1,3-propanediol; pH 5.0 to 9.0), 50 mM *N*-cyclohexyl-2-aminoethanesulfonate (pH 9.0 to 10.0), and 50 mM *N*-cyclohexyl-3-aminopropanesulfonate (pH 9.7 to 11.0) at 30 °C. The *K*_*m*_ and *k*_cat_ values were obtained from Hanes-Woolf plots and expressed as mean ± standard deviation from at least three independent experiments. Kinetic parameters of DesV and LigV were determined using the following substrate concentration ranges: DesV (0.4 μg/ml of protein) for SN, 0.25 to 5.0 μM; DesV (0.5 μg/ml of protein) for VN, 0.28 to 10.0 μM; LigV (20 μg/ml of protein) for SN, 25 to 3,200 μM; and LigV (0.2 μg/ml of protein) for VN, 0.25 to 5.0 μM.

### Sequence analyses of ALDHs

The deduced amino acid sequences of previously reported bacterial ALDH genes (Table S4) were obtained from NCBI database. To examine the presence of aromatic ALDH genes affiliated with the DesV, BzaA, and LigV/Vdh clusters in the living organism, a co-occurrence analysis of *desV, bzaA*, and *ligV* was conducted using the STRING database (http://string-db.org/) version 10^49^. Putative aromatic ALDH genes, the deduced amino acid sequences of which exhibited the highest similarity scores with *desV, bzaA*, and *ligV*, were picked up from 37 selected taxa of bacteria, eukaryotes, and archaea shown in Table S5. Construction of phylogenetic trees was done as described above. All the alignments are available upon request.

## Additional Information

**How to cite this article:** Kamimura, N. *et al*. A bacterial aromatic aldehyde dehydrogenase critical for the efficient catabolism of syringaldehyde. *Sci. Rep.* **7**, 44422; doi: 10.1038/srep44422 (2017).

**Publisher's note:** Springer Nature remains neutral with regard to jurisdictional claims in published maps and institutional affiliations.

## Supplementary Material

Supplementary Information

## Figures and Tables

**Figure 1:**
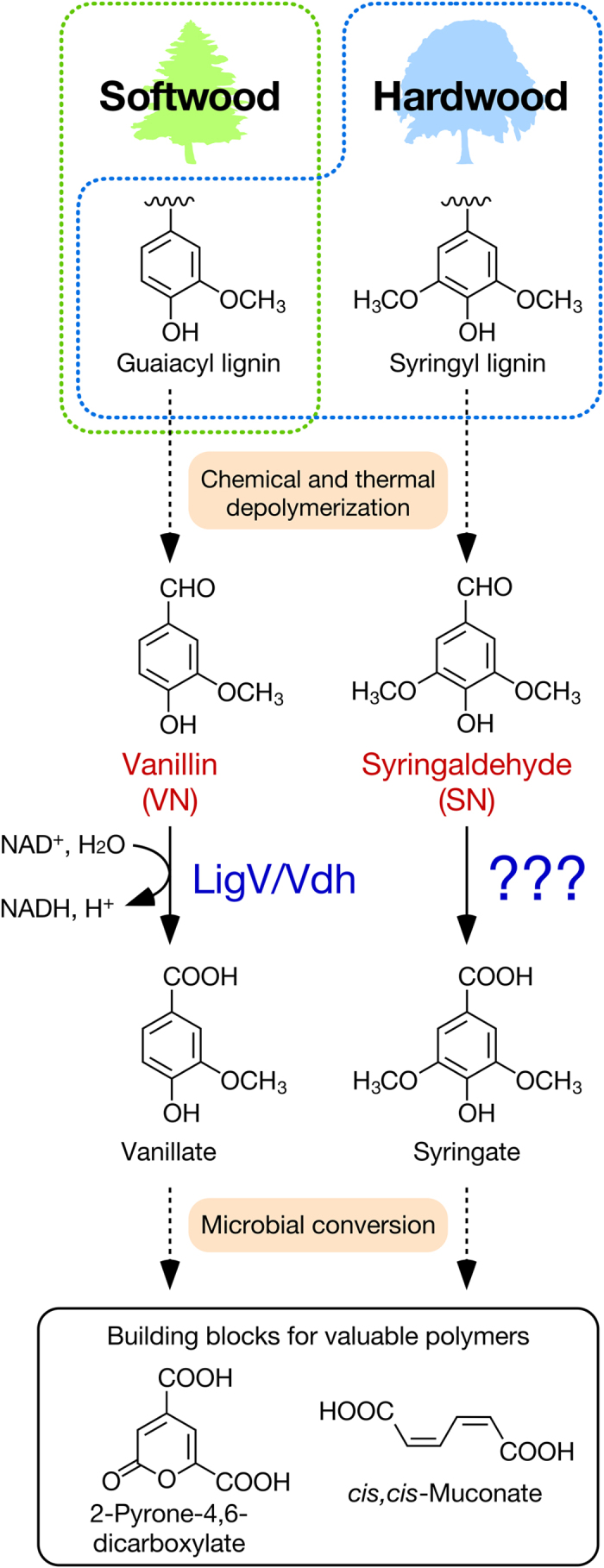
Lignin valorization through microbial catabolic functions. Vanillin (VN) and syringaldehyde (SN) are key intermediates for the production of industrially valued chemicals in microbial cell factories. VN and SN are initially oxidized to generate vanillate and syringate by VN and SN dehydrogenases, respectively. The VN dehydrogenase genes, *ligV* and *vdh*, are well characterized from various bacterial strains, whereas the SN dehydrogenase gene is not yet identified.

**Table 1:** Substrate range of the four putative SN dehydrogenases, LigV, BzaA, and BzaB.

Substrate	Specific activity (μmol·min^−1^·mg^−1^ of total protein)^a^						
	LigV	BzaA	BzaB	SLG_07270	SLG_07610	SLG_07790	SLG_28320 (DesV)
SN	0.36 ± 0.04 (8)	2.19 ± 0.52 (46)	0.12 ± 0.01 (23)	0.45 ± 0.06 (20)	0.52 ± 0.20 (28)	2.46 ± 0.58 (74)	1.80 ± 0.24 (85)
VN	1.80 ± 0.17 (41)	1.70 ± 0.55 (35)	0.09 ± 0.02 (17)	0.60 ± 0.07 (27)	0.33 ± 0.05 (18)	2.06 ± 0.58 (62)	1.57 ± 0.32 (74)
benzaldehyde	1.64 ± 0.50 (37)	2.02 ± 0.69 (42)	ND	0.19 ± 0.14 (8)	0.58 ± 0.15 (31)	2.05 ± 0.43 (62)	1.26 ± 0.62 (59)
*p*-hydroxybenzaldehyde	0.98 ± 0.32 (22)	4.17 ± 0.83 (87)	ND	0.10 ± 0.06 (5)	0.13 ± 0.02 (7)	2.27 ± 0.23 (68)	1.90 ± 0.80 (89)
protocatechualdehyde	1.68 ± 0.49 (38)	1.41 ± 0.30 (29)	ND	ND	ND	1.49 ± 0.22 (45)	1.24 ± 0.36 (58)
*m*-anisaldehyde	2.22 ± 0.53 (51)	0.42 ± 0.23 (9)	0.51 ± 0.04 (100)	2.25 ± 0.19 (100)	1.55 ± 0.29 (83)	0.49 ± 0.27 (15)	0.65 ± 0.24 (30)
veratraldehyde	0.13 ± 0.13 (3)	1.26 ± 0.27 (26)	0.39 ± 0.03 (77)	1.97 ± 0.14 (87)	0.77 ± 0.18 (41)	1.19 ± 0.37 (36)	1.38 ± 0.44 (65)
coniferyl aldehyde	0.05 ± 0.02 (1)	2.31 ± 0.41 (48)	0.26 ± 0.02 (52)	0.97 ± 0.19 (43)	0.12 ± 0.04 (6)	1.74 ± 0.62 (53)	2.12 ± 0.84 (100)
salicylaldehyde	1.04 ± 0.18 (24)	1.21 ± 0.11 (25)	ND	ND	0.04 ± 0.01 (2)	0.40 ± 0.07 (12)	2.12 ± 0.42 (100)
*m*-hydroxybenzaldehyde	4.39 ± 1.06 (100)	4.80 ± 1.20 (100)	0.10 ± 0.01 (21)	0.60 ± 0.08 (27)	1.86 ± 0.29 (100)	3.31 ± 0.66 (100)	2.09 ± 0.74 (99)

^a^Specific activities shown are values which have not been normalized to their expression levels. ND, not detected. Parentheses show the relative activities towards the substrates.

**Table 2:** Kinetic parameters of DesV and LigV.

Enzyme	Substrate	*V*_max_ (μmol·min^−1^·mg^−1^)	*k*_cat_ (s^−1^)	*K*_*m*_(μM)	*k*_cat_/*K*_*m*_(s^−1^·mM^−1^)
DesV	SN	1.08 ± 0.01	0.99 ± 0.01	0.48 ± 0.03	2,100 ± 93
	VN	0.85 ± 0.02	0.79 ± 0.01	0.47 ± 0.04	1,700 ± 140
LigV	SN	0.67 ± 0.01	0.59 ± 0.01	412 ± 3	1.4 ± 0.01
	VN	3.43 ± 0.04	3.03 ± 0.03	0.34 ± 0.01	8,800 ± 340
