# Supplementary material for: A bacterial aromatic aldehyde dehydrogenase critical for the efficient catabolism of syringaldehyde
Source: Sci Rep. 2017 Mar 15;7:44422. doi: 10.1038/srep44422 (PMC5353671; doi:10.1038/srep44422)
Supplement: Supplementary Information [file srep44422-s1.pdf]

Supplementary information

A bacterial aromatic aldehyde dehydrogenase critical for the efficient catabolism of syringaldehyde

Naofumi Kamimura,[1] Takayuki Goto,[1] Kenji Takahashi,[1] Daisuke Kasai,[1]
Yuichiro Otsuka,[2] Masaya Nakamura,[2] Yoshihiro Katayama,[3]
Masao Fukuda,[1] and Eiji Masai[1*]

[1)] *Department of Bioengineering, Nagaoka University of Technology, Nagaoka, Niigata 940-2188, Japan*
[2)] *Forestry and Forest Products Research Institute, Tsukuba, Ibaraki 305-8687, Japan*
[3)] *College of Bioresource Sciences, Nihon University, Fujisawa, Kanagawa 252-0880, Japan*

[*]Corresponding author:
Eiji Masai
Department of Bioengineering, Nagaoka University of Technology, Nagaoka, Niigata 940-2188, Japan
E-mail: emasai@vos.nagaokaut.ac.jp

Contents list

Supplementary tables: Table S1–S7
Supplementary figures: Fig. S1–S10
References for Supplementary information

Table S1. Twenty putative ALDH genes and three previously characterized aromatic ALDH genes in *Sphingobium* sp. SYK-6

Locus tag (gene)	Accession No.	Size (a.a.)	Product	Similar ALDH reported previously[a]			
				Gene	Strain	Product	Identity (%)
SLG_07060 (*ligV*)	BAK65381	480	vanillin dehydrogenase	*vdh*	*Pseudomonas fluorescens* biovar V, AN103	vanillin dehydrogenase	55.4
SLG_07270	BAK65402	494	NAD^+-dependent aldehyde dehydrogenase	*dhaS*	*Bacillus subtilis* subsp. subtilis 168	3-hydroxypropionaldehyde dehydrogenase	37.3
SLG_07610	BAK65436	486	NAD^+-dependent aldehyde dehydrogenase	*cymC*	*Pseudomonas putida* F1	*p*-cumic aldehyde dehydrogenase	39.6
SLG_07790	BAK65454	483	aminobutyraldehyde dehydrogenase	*cymC*	*Pseudomonas putida* F1	*p*-cumic aldehyde dehydrogenase	38.1
SLG_09400	BAK65615	484	NAD^+-dependent aldehyde dehydrogenase	*betB1*	*Sinorhizobium meliloti* 1021	betaine aldehyde dehydrogenase	30.2
SLG_09510	BAK65626	499	NAD^+-dependent aldehyde dehydrogenase	*cymC*	*Pseudomonas putida* F1	*p*-cumic aldehyde dehydrogenase	48.9
SLG_09920	BAK65667	472	*N*-succinylglutamate 5-semialdehyde dehydrogenase	*aruD*	*Pseudomonas aeruginosa* PAO1	succinylglutamate 5-semialdehyde dehydrogenase	50.7
SLG_11410	BAK65816	498	NAD^+-dependent aldehyde dehydrogenase	*cymC*	*Pseudomonas putida* F1	*p*-cumic aldehyde dehydrogenase	45.7
SLG_12020	BAK65877	475	NAD^+-dependent aldehyde dehydrogenase	*dhaS*	*Bacillus subtilis* subsp. subtilis 168	3-hydroxypropionaldehyde dehydrogenase	39.6
SLG_12190	BAK65894	487	succinate-semialdehyde dehydrogenase	*gabD*	*Pseudomonas putida* KT2440	succinate-semialdehyde dehydrogenase	53.5
SLG_12800	BAK65955	476	putative succinate-semialdehyde dehydrogenase	*araE*	*Azospirillum brasilense* ATCC29145	α-ketoglutaric semialdehyde dehydrogenase	48.4
SLG_18210	BAK66496	521	α-ketoglutaric semialdehyde dehydrogenase	ACIAD0131	*Acinetobacter* sp. ADP1	α-ketoglutaric semialdehyde dehydrogenase	47.4
SLG_20400	BAK66715	453	aldehyde dehydrogenase	*alkH*	*Pseudomonas oleovorans* GPo1	aldehyde dehydrogenase	41.0
SLG_27210	BAK67396	508	NAD^+-dependent aldehyde dehydrogenase	*ald1*	*Acinetobacter* sp. M-1	long-chain-aldehyde dehydrogenase	70.3
SLG_27910 (*bzaA*)	BAK67466	472	benzaldehyde-derivatives dehydrogenase	*patD*	*Escherichia coli* K-12	γ-aminobutyraldehyde dehydrogenase	38.7
SLG_27920 (*bzaB*)	BAK67467	489	benzaldehyde-derivatives dehydrogenase	*betB1*	*Sinorhizobium meliloti* 1021	betaine aldehyde dehydrogenase	37.6
SLG_28150	BAK67490	501	methylmalonate-semialdehyde dehydrogenase	*bauC*	*Pseudomonas aeruginosa* PAO1	3-oxopropanoate dehydrogenase	58.0
SLG_28320 (*desV*)	BAK67507	490	NAD^+-dependent aldehyde dehydrogenase	*cymC*	*Pseudomonas putida* F1	*p*-cumic aldehyde dehydrogenase	39.2
SLG_31150	BAK67790	483	succinate-semialdehyde dehydrogenase	*gabD*	*Pseudomonas putida* KT2440	succinate-semialdehyde dehydrogenase	60.4
SLG_32240	BAK67899	476	*N*-succinylglutamate 5-semialdehyde dehydrogenase	*aruD*	*Pseudomonas aeruginosa* PAO1	succinylglutamate 5-semialdehyde dehydrogenase	39.4
SLG_34940	BAK68169	479	putative succinate-semialdehyde dehydrogenase	*araE*	*Azospirillum brasilense* ATCC29145	α-ketoglutaric semialdehyde dehydrogenase	55.1
SLG_38120	BAK68487	493	phenylacetaldehyde dehydrogenase	*dhaS*	*Bacillus subtilis* subsp. subtilis 168	3-hydroxypropionaldehyde dehydrogenase	49.0
SLG_p_00680	BAK68656	506	NAD^+-dependent aldehyde dehydrogenase	*aldB*	*Escherichia coli* K-12	aldehyde dehydrogenase B	65.4

[a]Accession numbers and references are shown in Table S4.

Table S2. Expression levels of ALDH genes of *Sphingobium* sp. SYK-6 in *E. coli*

Locus tag (gene)	Intensities of bands		Expression levels	Normalization values
	ALDH	whole proteins	(Ratio of ALDH/whole proteins)	(LigV = 1.0)
SLG_07060 (*ligV*)	359860032	1319102698	0.27	1.00
SLG_07270	460016606	1721456966	0.27	0.98
SLG_07610	466285120	1442608286	0.32	1.18
SLG_07790	386997376	1354605848	0.29	1.05
SLG_09400	379634421	1628950984	0.23	0.85
SLG_09510	483421903	1823055183	0.27	0.97
SLG_09920	424695341	1536471516	0.28	1.01
SLG_11410	370585208	2085443232	0.18	0.65
SLG_12020	588296858	1738536259	0.34	1.24
SLG_12190	223056299	905216566	0.25	0.90
SLG_12800	138234622	853718418	0.16	0.59
SLG_18210	340145167	1024693973	0.33	1.22
SLG_20400	491817990	1423208131	0.35	1.27
SLG_27210	344233696	1210247689	0.28	1.04
SLG_27910 (*bzaA*)	259989124	805195112	0.32	1.18
SLG_27920 (*bzaB*)	478132646	1777190172	0.27	0.99
SLG_28150	515831152	1738480547	0.30	1.09
SLG_28320 (*desV*)	444415320	1626411864	0.27	1.00
SLG_31150	190575850	848707454	0.22	0.82
SLG_32240	0	1421923677	0.00	0.00
SLG_34940	0	962775420	0.00	0.00
SLG_38120	120831483	1242630301	0.10	0.36
SLG_p_00680	494333213	1513990549	0.33	1.20

Table S3. Kinetic parameters of aromatic ALDHs for VN conversion

Enzyme	Organism	V_{max} (μmol·min^{-1}·mg^{-1})	k_{cat} (s^{-1})	K_m (μM)	k_{cat}/K_m (s^{-1}·mM^{-1})	Reference
LigV	*Sphingobium* sp. SYK-6	3.43	3.03	0.34	8,800	This study
DesV	*Sphingobium* sp. SYK-6	0.85	0.79	0.47	1,700	This study
Vdh	*Corynebacterium glutamicum*	–	21.03	26.31	800	(1)
Vdh	*Micrococcus* sp. TA1	0.39	–	7	–	(2)
Vdh	*Burkholderia cepacia* TM1	1.3	–	4	–	(2)
HcaB	*Acinetobacter* sp. ADP1	–	18	44	410	(3)
ALDH1A1	*Homo sapiens*	–	–	0.86	–	(4)
ALDH3A1	*Homo sapiens*	–	–	152	–	(4)

Table S4. Bacterial ALDH genes used for phylogenetic analysis

Abbreviation	Gene	Accession No.	Strain	Product	Reference
Ald1_AsM1	*ald1*	BAB11888	*Acinetobacter* sp. M-1	long-chain-aldehyde dehydrogenase	(5)
AldA_EcK12	*aldA*	AAA23427	*Escherichia coli* K-12	lactaldehyde dehydrogenase	(6)
AldB_EcK12	*aldB*	AAC36939	*Escherichia coli* K-12	aldehyde dehydrogenase B	(7)
AldH_AnpAO1	*aldH*	CAD47897	*Arthrobacter nicotinovorans* pAO1	aldehyde dehydrogenase	(8)
AldH_VhB392	*aldH*	AAA89078	*Vibrio harveyi* B392	fatty aldehyde dehydrogenase	(9)
AldhT_GsSIC1	*aldHT*	BAA02975	*Geobacillus stearothermophilus* SIC1	aldehyde dehydrogenase	(10)
AldhT_SsP2	*aldhT*	AAK43220	*Sulfolobus solfataricus* P2	2,5-dioxopentanoate dehydrogenase	(11)
AlkH_PoGPo1	*alkH*	CAB54053	*Pseudomonas oleovorans* GPo1	aldehyde dehydrogenase	(12)
AmnC_PsAP3	*amnC*	BAB03533	*Pseudomonas* sp. AP-3	2-aminomuconic 6-semialdehyde dehydrogenase	(13)
AraE_AbATCC29145	*araE*	BAE94276	*Azospirillum brasilense* ATCC29145	α-ketoglutaric semialdehyde dehydrogenase	(14)
AreC_AsADP1	*areC*	AAD34025	*Acinetobacter* sp. ADP1	benzaldehyde dehydrogenase	(15)
AruD_PaPAO1	*aruD*	AAC46012	*Pseudomonas aeruginosa* PAO1	succinylglutamate 5-semialdehyde dehydrogenase	(16)
AstD_EcK12	*astD*	AAC74816	*Escherichia coli* K-12	*N*-succinylglutamate 5-semialdehyde dehydrogenase	(17)
BauC_PaPAO1	*bauC*	AAG03520	*Pseudomonas aeruginosa* PAO1	3-oxopropanoate dehydrogenase	(18)
BetB_EcK12	*betB*	AAB18038	*Escherichia coli* K-12	betaine aldehyde dehydrogenase	(19)
BetB1_Sm1021	*betB1*	CAC45520	*Sinorhizobium meliloti* 1021	betaine aldehyde dehydrogenase	(20)
BoxD_AeKB740	*boxD*	AAN39373	*Azoarcus evansii* KB740	3,4-dehydroadipyl-CoA semialdehyde dehydrogenase	(21)
CalB_PsHR199	*calB*	CAA06926	*Pseudomonas* sp. HR199	coniferyl aldehyde dehydrogenase	(22)
CarD_GfFKMC1995	*carD*	CCA63431	*Gibberella fujikuroi* FKMC1995	β-apo-4'-carotenal dehydrogenase	(23)
ChnE_AsNCIMB9871	*chnE*	BAA86294	*Acinetobacter* sp. NCIMB 9871	6-oxohexanoate dehydrogenase	(24)
ChnE_RsPhi2	*chnE*	AAN37492	*Rhodococcus* sp. Phi2	6-oxohexanoate dehydrogenase	(25)
CymC_PpF1	*cymC*	AAB62298	*Pseudomonas putida* F1	*p*-cumic aldehyde dehydrogenase	(26)
DhaS_Bs168	*DhaS*	AAB84440	*Bacillus subtilis* subsp. subtilis 168	3-hydroxypropionaldehyde dehydrogenase	(27)
DoeC_He1H9	*doeC*	CBV43546	*Halomonas elongata* 1H9	aspartate-semialdehyde dehydrogenase	(28)
FeaB_EcK12	*feaB*	AAC74467	*Escherichia coli* K-12	phenylacetaldehyde dehydrogenase	(29)
GabD_EcK12	*gabD*	BAA16524	*Escherichia coli* K-12	succinic semialdehyde dehydrogenase	(30)
GabD_PpKT2440	*gabD*	AAN65845	*Pseudomonas putida* KT2440	succinate-semialdehyde dehydrogenase	(31)
GabD1_SpMGAS1882	*gabD1*	AFC68023	*Streptococcus pyogenes* MGAS1882	succinic semialdehyde dehydrogenase	(32)
GapN_SbJB1	*gapN*	BAE46989	*Streptococcus bovis* JB1	glyceraldehyde-3-phosphate dehydrogenase	(33)
GapN_TtKRA1	*gapN*	CAA71651	*Thermoproteus tenax* KRA 1	glyceraldehyde-3-phosphate dehydrogenase	(34)
GbsA_Bs168	*gbsA*	AAC44364	*Bacillus subtilis* JH642	betaine aldehyde dehydrogenase	(35)
HcaB_AsADP1	*hcaB*	CAG68567	*Acinetobacter* sp. ADP1	hydroxybenzaldehyde dehydrogenase	(3)
HpaE_EcW	*hpaE*	AFH14227	*Escherichia coli* W	5-carboxymethyl-2-hydroxymuconate semialdehyde dehydrogenase	(36)
HpcC_EcC	*hpcC*	CAA57102	*Escherichia coli* C	5-carboxymethyl-2-hydroxymuconate semialdehyde dehydrogenase	(37)
IolA_Bs168	*iolA*	BAA21609	*Bacillus subtilis* subsp. subtilis 168	methylmalonate semialdehyde dehydrogenase	(38)
KGSDH_AsADP1	ACIAD0131	Q6FFQ0	*Acinetobacter* sp. ADP1	α-ketoglutaric semialdehyde dehydrogenase	(39)
KGSDH_HvDS2	HVO_B0039	ADE01373	*Haloferax volcanii* DS2	α-ketoglutarate semialdehyde dehydrogenase	(40)
MdlD_PpATCC12633	*mdlD*	AAO23020	*Pseudomonas putida* ATCC 12633	benzaldehyde dehydrogenase	(41)
MmsA_PaPAO	*mmsA*	AAA25891	*Pseudomonas aeruginosa* PAO	methylmalonate semialdehyde dehydrogenase	(42)
NahF_PfBS202	*nahF*	ACQ63494	*Pseudomonas fluorescens* PC20	salicylaldehyde dehydrogenase	(43)
PatD_EcK12	*patD*	AAC74526	*Escherichia coli* K-12	γ-aminobutyraldehyde dehydrogenase	(44)
PchA_PpNCIMB9866	*pchA*	AGO01127	*Pseudomonas putida* NCIMB 9866	*p*-hydroxybenzaldehyde dehydrogenase	(45)
PcuC_PmKR1	*pcuC*	AAM92190	*Pseudomonas mendocina* KR1	*p*-hydroxybenzaldehyde dehydrogenase	(46)
PedI_PpU	*pedI*	ABR57228	*Pseudomonas putida* U	phenylacetaldehyde dehydrogenase	(47)
PhdK_NsKP7	*phdK*	BAA31236	*Nocardioides* sp. KP7	2-carboxybenzaldehyde dehydrogenase	(48)
PhnI_AfAFK2	*phnI*	BAA76329	*Alcaligenes faecalis* AFK2	2-carboxybenzaldehyde dehydrogenase	(49)
PP1948_PpKT2440	PP_1948	AAN67564	*Pseudomonas putida* KT2440	benzaldehyde dehydrogenase	(50)

PP0545_PpKT2440	PP_0545	AAN66172	*Pseudomonas putida* KT2440	aldehyde dehydrogenase	(50)
PP2680_PpKT2440	PP_2680	AAN68288	*Pseudomonas putida* KT2440	aldehyde dehydrogenase	(50)
PP3151_PpKT2440	PP_3151	AAN68759	*Pseudomonas putida* KT2440	succinate semialdehyde dehydrogenase	(50)
PP5120_PpKT2440	PP_5120	AAN70685	*Pseudomonas putida* KT2440	coniferyl aldehyde dehydrogenase	(50)
PP5258_Pp_KT2440	PP_5258	AAN70823	*Pseudomonas putida* KT2440	L-piperidine-6-carboxylate dehydrogenase	(50)
PuuC_EcK12	*puuC*	AAA23428	*Escherichia coli* K-12	aldehyde dehydrogenase	(51)
RocA_BlDSM13	*rocA*	AAU39332	*Bacillus licheniformis* DSM 13	1-pyrroline-5-carboxylate dehydrogenase	(52)
Sad_EcK12	*sad*	AAC74598	*Escherichia coli* K-12	succinate semialdehyde dehydrogenase	(53)
SafD_NcMED92	*safD*	EAR61922	*Neptuniibacter caesariensis* MED92	sulfoacetaldehyde dehydrogenase	(54)
StyD_PpPf-5	*styD*	CAB06826	*Pseudomonas fluorescens* ST	phenylacetaldehyde dehydrogenase	(55)
TsaD_CtT2	*tsaD*	AAC44808	*Comamonas testosteroni* T-2	toluenesulfonate aldehyde dehydrogenase	(56)
TynC_PpU	*tynC*	ADA63511	*Pseudomonas putida* U	4-hydroxyphenylacetaldehyde dehydrogenase	(57)
Vdh_AsATCC39116	*vdh*	AFY98904	*Amycolatopsis* sp. ATCC 39116	vanillin dehydrogenase	(58)
Vdh_CgATCC13032	*vdh*	BAC00062	*Corynebacterium glutamicum* ATCC 13032	vanillin dehydrogenase	(1)
Vdh_PfAN103	*vdh*	CAA73503	*Pseudomonas fluorescens* biovar V, AN103	vanillin dehydrogenase	(59)
Vdh_PpKT2440	*vdh*	AAN68961	*Pseudomonas putida* KT2440	vanillin dehydrogenase	(60)
Vdh_PpWCS358	*vdh*	CAA75076	*Pseudomonas putida* WCS358	vanillin dehydrogenase	(61)
Vdh_PsHR199	*vdh*	CAA72286	*Pseudomonas* sp. HR199	vanillin dehydrogenase	(62)
VdH_RaI24	*vdh*	AAY98503	*Rhodococcus aetherivorans* I24	vanillin dehydrogenase	(63)
Vdh_RjRHA1	*vdh*	ABG94789	*Rhodococcus jostii* RHA1	vanillin dehydrogenase	(64)
XylC_Ppmt-2	*xylC*	AAA66218	*Pseudomonas putida* mt-2	benzaldehyde dehydrogenase	(65)
XylG_Ppmt-2	*xylG*	AAA26053	*Pseudomonas putida* mt-2	2-hydroxymuconic semialdehyde dehydrogenase	(65)

Table S5. Putative ALDHs showing high similarities with DesV, BzaA, and LigV from bacteria, eukaryotes, and archaea

Abbreviations	Searched ALDHs	STRING Organism (Taxonomy rank, number of taxa)	Domain	Kingdom	Phylum	Class	Order	Family	Genus	Organism	Accession No.	Locus tag	STRING simirality score (bits)	Identity	Cluster	Definition
DV_GP_Entero	DesV	Enterobacteriaceae (Family, 83 taxa)	Bacteria		Proteobacteria	Gammaproteobacteria	Enterobacterales	Enterobacteriaceae		Enterobacter cancerogenus ATCC 35316	EFC57858	ENTCAN_05297	310	39.0% (vs DesV)		aldehyde dehydrogenase (NAD) family protein
DV_GP_Altero	DesV	Alteromonadales (Order, 41 taxa)	Bacteria		Proteobacteria	Gammaproteobacteria	Alteromonadales			Colwellia psychrerythraea 34H	AAZ28154	CPS_0096	346	36.4% (vs DesV)		betaine aldehyde dehydrogenase
DV_GP_Pseudo	DesV	Pseudomonadales (Order, 38 taxa)	Bacteria		Proteobacteria	Gammaproteobacteria	Pseudomonadales			Pseudomonas fluorescens F113	AEV62579	PSF113_2572	439	46.8% (vs DesV)	DesV	aldehyde dehydrogenase
DV_GP_Vibrio	DesV	Vibrionales (Order, 29 taxa)	Bacteria		Proteobacteria	Gammaproteobacteria	Vibrionales			Vibrio shilonii AK1	EDL55511	VSAK1_23264	300	32.7% (vs DesV)		NAD-dependent aldehyde dehydrogenase
DV_GP_Oceno	DesV	Oceanospirillales (Order, 13 taxa)	Bacteria		Proteobacteria	Gammaproteobacteria	Oceanospirillales			Neptuniibacter caesariensis	EAR60903	MED92_01851	367	42.0% (vs DesV)		aldehyde dehydrogenase
DV_GP_Xantho	DesV	Xanthomonadaceae (Family, 10 taxa)	Bacteria		Proteobacteria	Gammaproteobacteria	Xanthomonadales	Xanthomonadaceae		Xanthomonas oryzae pv. oryzicola BLS256	AEQ95139	XOC_0933	655	64.9% (vs DesV)	DesV	aldehyde dehydrogenase
DV_AP_Rhizo	DesV	Rhizobiales (Order, 67 taxa)	Bacteria		Proteobacteria	Alphaproteobacteria	Rhizobiales			Mesorhizobium loti MAFF303099	BAB53154	mlr6969	346	39.9% (vs DesV)		aldehyde dehydrogenase
DV_AP_Rhodo	DesV	Rhodobacterales (Order, 52 taxa)	Bacteria		Proteobacteria	Alphaproteobacteria	Rhodobacterales			Pelagibaca bermudensis HTCC2601	EAU48723	R2601_04083	368	41.5% (vs DesV)	DesV	aldehyde dehydrogenase
DV_AP_Ricke	DesV	Rickettsiales (Order, 23 taxa)	Bacteria		Proteobacteria	Alphaproteobacteria	Rickettsiales			Rickettsia akari str. Hartford	ABV74960	A1C_03370	239	31.0% (vs DesV)		succinate semialdehyde dehydrogenase
DV_AP_Sp_NA	DesV	Novosphingobium aromaticivorans (Species, 1)	Bacteria		Proteobacteria	Alphaproteobacteria	Sphingomonadales	Sphingomonadaceae	Novosphingobium	Novosphingobium aromaticivorans DSM 12444	ABD27237	Saro_2801	376	44.1% (vs DesV)	DesV	aldehyde dehydrogenase
DV_AP_Sp_SC	DesV	Sphingobium chlorophenolicum (Species, 1)	Bacteria		Proteobacteria	Alphaproteobacteria	Sphingomonadales	Sphingomonadaceae	Sphingobium	Sphingobium chlorophenolicum L-1	AEG50859	Sphch_3249	458	50.0% (vs DesV)	DesV	Betaine-aldehyde dehydrogenase
DV_AP_Sp_SJ	DesV	Sphingobium japonicum (Species, 1)	Bacteria		Proteobacteria	Alphaproteobacteria	Sphingomonadales	Sphingomonadaceae	Sphingobium	Sphingobium japonicum UT26S	BAI96225	SJA_C1-13910	320	36.2% (vs DesV)		NAD-dependent aldehyde dehydrogenase
DV_AP_Sp_SW	DesV	Sphingomonas wittichii (Species, 1)	Bacteria		Proteobacteria	Alphaproteobacteria	Sphingomonadales	Sphingomonadaceae	Sphingobium	Sphingomonas wittichii RW1	ABQ68339	Swit_1979	462	48.8% (vs DesV)	DesV	aldehyde dehydrogenase
DV_AP_Sp_CB	DesV	Citromicrobium bathyomarinum (Species, 1)	Bacteria		Proteobacteria	Alphaproteobacteria	Sphingomonadales	Sphingomonadaceae	Citromicrobium	Citromicrobium bathyomarinum JL354	ZP_06861512	CbatJ_010100007826	426	40.9% (vs DesV)	DesV	putative aldehyde dehydrogenase
DV_AP_Sp_ZM	DesV	Zymomonas mobilis (Species, 2)	Bacteria		Proteobacteria	Alphaproteobacteria	Sphingomonadales	Sphingomonadaceae	Zymomonas	Zymomonas mobilis subsp. pomaceae ATCC 29192	AEI37986	Zymop_1090	249	31.7% (vs DesV)		Succinate-semialdehyde dehydrogenase
DV_AP_Sp_Ery	DesV	Erythrobacter (Species, 3 taxa)	Bacteria		Proteobacteria	Alphaproteobacteria	Sphingomonadales	Erythrobacteraceae	Erythrobacter	Erythrobacter sp. SD-21	EDL48349	ED21_22573	281	38.4% (vs DesV)		aldehyde dehydrogenase
DV_AP_Rhos	DesV	Rhodospirillales (Order, 15 taxa)	Bacteria		Proteobacteria	Alphaproteobacteria	Rhodospirillales			Magnetospirillum magneticum AMB-1	BAE51397	amb2593	312	40.2% (vs DesV)		NAD-dependent aldehyde dehydrogenase
DV_AP_Cau	DesV	Caulobacteraceae (Family, 9 taxa)	Bacteria		Proteobacteria	Alphaproteobacteria	Caulobacterales	Caulobacteraceae		Caulobacter crescentus NA1000	ACL93891	CC_0419	325	39.5% (vs DesV)		aldehyde dehydrogenase
DV_BP_Bur	DesV	Burkholderiales (Order, 66 taxa)	Bacteria		Proteobacteria	Betaproteobacteria	Burkholderiales			Paraburkholderia phymatum STM815	ACC72358	Bphy_3198	481	46.7% (vs DesV)	DesV	Aldehyde Dehydrogenase
DV_BP_Nei	DesV	Neisseriaceae (Family, 23 taxa)	Bacteria		Proteobacteria	Betaproteobacteria	Neisseriales	Neisseriaceae		Pseudogulbenkiania ferrooxidans 2002	EEG09632	FuraDRAFT_0648	347	39.4% (vs DesV)		Aldehyde Dehydrogenase
DV_BP_Rho	DesV	Rhodocyclaceae (Family, 6 taxa)	Bacteria		Proteobacteria	Betaproteobacteria	Rhodocyclales	Rhodocyclaceae		Aromatoleum aromaticum EbN1	CAI08743	ebA4621	349	43.3% (vs DesV)		putative betaine aldehyde dehydrogenase (BADH) oxidoreductase
DV_BP_Nit	DesV	Nitrosomonadaceae (Family, 5 taxa)	Bacteria		Proteobacteria	Betaproteobacteria	Nitrosomonadales	Nitrosomonadaceae		Nitrosomonas sp. AL212	ADZ27487	NAL212_2697	244	33.0% (vs DesV)		Betaine-aldehyde dehydrogenase
DV_BP_Met	DesV	Methylophilales (Order, 5 taxa)	Bacteria		Proteobacteria	Betaproteobacteria	Methylophilales			Methylotenera versatilis 301	ADI29454	M301_1070	300	37.7% (vs DesV)		Betaine-aldehyde dehydrogenase
DV_DelEpsP	DesV	deltaepsilon subdivisions (Class, 91 taxa)	Bacteria		Proteobacteria	Delta/Epsilon subdivision (no lank)				Pelobacter carbinolicus DSM 2380	ABA88944	Pcar_1701	358	43.8% (vs DesV)		omega-oxo-fatty acid dehydrogenase
DV_Firm_Baci	DesV	Bacilli (Class, 201 taxa)	Bacteria		Firmicutes	Bacilli				Bacillus megaterium QM B1551	ADE69067	BMQ_2038	360	41.8% (vs DesV)		aldehyde dehydrogenase (NAD) family protein
DV_Firm_Clos	DesV	Clostridia (Class, 174 taxa)	Bacteria		Firmicutes	Clostridia				Clostridium beijerinckii NCIMB 8052	ABR32911	Cbei_0727	332	38.0% (vs DesV)		aldehyde dehydrogenase
DV_Actino	DesV	Actinobacteria class (Class, 181 taxa)	Bacteria		Actinobacteria	Actinobacteria				Rhodococcus equi 103S	CBH49678	REQ_36910	464	48.4% (vs DesV)	DesV	aldehyde dehydrogenase
DV_Bacteroid	DesV	BacteroidetesChlorobi group (Phylum, 145 taxa)	Bacteria		Bacteroidetes and Chlorobi (no rank)					Chryseobacterium gleum ATCC 35910	EFK35157	HMPREF0204_14226	322	41.3% (vs DesV)		aldehyde dehydrogenase (NAD) family protein
DV_Cyano	DesV	Cyanobacteria (Phylum, 57 taxa)	Bacteria		Cyanobacteria					Synechococcus sp. JA-2-3B'a(2-13)	ABD01699	CYB_0715	348	39.6% (vs DesV)		aldehyde dehydrogenase family protein
DV_Deino	DesV	Deinococci (Class, 15 taxa)	Bacteria		Deinococcus-Thermus	Deinococci				Deinococcus radiodurans R1	AAF12231	DR_A0126	342	42.4% (vs DesV)		aldehyde dehydrogenase
DV_Chloro	DesV	Chloroflexi (Phylum, 14 taxa)	Bacteria		Chloroflexi					Ktedonobacter racemifer DSM 44963	EFH82944	Krac_3840	426	42.5% (vs DesV)	DesV	aldehyde dehydrogenase
DV_Euc_Meta	DesV	Metazoa (Kingdom, 89 taxa)	Eukaryotes	Metazoa						Drosophila virilis	EDW71313	Dvir_GJ16294	315	34.2% (vs DesV)		uncharacterized protein
DV_Euc_Fung	DesV	Fungi (Kingdom, 85 taxa)	Eukaryotes	Fungi						Nectria haematococca mpVI 77-13-4	EEU37952	NechaP98069	342	38.6% (vs DesV)		hypothetical protein NECHADRAFT_98069
DV_Euc_Virid	DesV	Viridiplantae (Kingdom, 23 taxa)	Eukaryotes	Viridiplantae						Vitis vinifera	XP_002281984	LOC100250859	339	37.8% (vs DesV)		betaine aldehyde dehydrogenase 1
DV_Euc_Alveo	DesV	Alveolata (Kingdom, 16 taxa)	Eukaryotes	Alveolata (no rank)						Perkinsus marinus ATCC 50983	XP_002788841	Pmar_PMAR004301	301	35.4% (vs DesV)		aldehyde dehydrogenase
DV_Arc_Eurya	DesV	Euryarchaeota (Phylum, 79 taxa)	Archaea		Euryarchaeota					Methanosaeta thermophila PT	ABK14188	Mthe_0395	295	35.2% (vs DesV)		succinate semialdehyde dehydrogenase
DV_Arc_Themo	DesV	Thermoprotei (Class, 30 taxa)	Archaea		Crenarchaeota	Thermoprotei				Vulcanisaeta distributa DSM 14429	ADN51031	Vdis_1653	303	34.0% (vs DesV)		aldehyde dehydrogenase
BA_GP_Entero	BzaA	Enterobacteriaceae (Family, 83 taxa)	Bacteria		Proteobacteria	Gammaproteobacteria	Enterobacterales	Enterobacteriaceae		Enterobacter aerogenes	BAD88608	MoaM	317	38.4% (vs BzaA)		aldehyde dehydrogenase
BA_GP_Altero	BzaA	Alteromonadales (Order, 41 taxa)	Bacteria		Proteobacteria	Gammaproteobacteria	Alteromonadales			Pseudoalteromonas atlantica T6c	ABG38685	Patl_0153	479	46.6% (vs BzaA)	BzaA	Betaine-aldehyde dehydrogenase
BA_GP_Pseudo	BzaA	Pseudomonadales (Order, 38 taxa)	Bacteria		Proteobacteria	Gammaproteobacteria	Pseudomonadales			Pseudomonas fluorescens SBW25	CAY49320	PFLU_3090	431	46.8% (vs BzaA)	BzaA	aldehyde dehydrogenase
BA_GP_Vibrio	BzaA	Vibrionales (Order, 29 taxa)	Bacteria		Proteobacteria	Gammaproteobacteria	Vibrionales			Vibrio mimicus MB451	EEY38629	VII_002389	404	43.2% (vs BzaA)	BzaA	aldehyde dehydrogenase
BA_GP_Oceno	BzaA	Oceanospirillales (Order, 13 taxa)	Bacteria		Proteobacteria	Gammaproteobacteria	Oceanospirillales			Marinomonas sp. MWYL1	ABR70758	Mmwyl1_1834	436	46.3% (vs BzaA)	BzaA	aldehyde dehydrogenase
BA_GP_Xantho	BzaA	Xanthomonadaceae (Family, 10 taxa)	Bacteria		Proteobacteria	Gammaproteobacteria	Xanthomonadales	Xanthomonadaceae		Stenotrophomonas maltophilia K279a	CAQ45735	Smlt2238	262	35.2% (vs BzaA)		Betaine-aldehyde dehydrogenase
BA_AP_Rhizo	BzaA	Rhizobiales (Order, 67 taxa)	Bacteria		Proteobacteria	Alphaproteobacteria	Rhizobiales			Parvibaculum lavamentivorans DS-1	ABS61715	Plav_0092	561	57.2% (vs BzaA)	BzaA	aldehyde dehydrogenase
BA_AP_Rhodo	BzaA	Rhodobacterales (Order, 52 taxa)	Bacteria		Proteobacteria	Alphaproteobacteria	Rhodobacterales			Paracoccus sp. TRP	WP_010395468	PaTRP_010100008861	510	55.3% (vs BzaA)	BzaA	aldehyde dehydrogenase
BA_AP_Ricke	BzaA	Rickettsiales (Order, 23 taxa)	Bacteria		Proteobacteria	Alphaproteobacteria	Rickettsiales			Rickettsia akari str. Hartford	ABV74960	A1C_03370	267	32.6% (vs BzaA)		succinate semialdehyde dehydrogenase
BA_AP_Sp_NA	BzaA	Novosphingobium aromaticivorans (Species, 1)	Bacteria		Proteobacteria	Alphaproteobacteria	Sphingomonadales	Sphingomonadaceae	Novosphingobium	Novosphingobium aromaticivorans DSM 12444	ABD26132	Saro_1692	520	55.4% (vs BzaA)	BzaA	aldehyde dehydrogenase
BA_AP_Sp_SC	BzaA	Sphingobium chlorophenolicum (Species, 1)	Bacteria		Proteobacteria	Alphaproteobacteria	Sphingomonadales	Sphingomonadaceae	Sphingobium	Sphingobium chlorophenolicum L-1	AEG50983	Sphch_3387	462	48.6% (vs BzaA)	BzaA	aldehyde dehydrogenase
BA_AP_Sp_SJ	BzaA	Sphingobium japonicum (Species, 1)	Bacteria		Proteobacteria	Alphaproteobacteria	Sphingomonadales	Sphingomonadaceae	Sphingobium	Sphingobium japonicum UT26S	BAI98864	SJA_C2-05010	385	43.3% (vs BzaA)	BzaA	putative betaine-aldehyde dehydrogenase
BA_AP_Sp_SW	BzaA	Sphingomonas wittichii (Species, 1)	Bacteria		Proteobacteria	Alphaproteobacteria	Sphingomonadales	Sphingomonadaceae	Sphingobium	Sphingomonas wittichii RW1	ABQ68424	Swit_2065	650	64.1% (vs BzaA)	BzaA	aldehyde dehydrogenase
BA_AP_Sp_CB	BzaA	Citromicrobium bathyomarinum (Species, 1)	Bacteria		Proteobacteria	Alphaproteobacteria	Sphingomonadales	Sphingomonadaceae	Citromicrobium	Citromicrobium sp. JL477	ALG60477	WG74_06205	461	49.3% (vs BzaA)	BzaA	aldehyde dehydrogenase
BA_AP_Sp_ZM	BzaA	Zymomonas mobilis (Species, 2)	Bacteria		Proteobacteria	Alphaproteobacteria	Sphingomonadales	Sphingomonadaceae	Zymomonas	Zymomonas mobilis subsp. pomaceae ATCC 29192	AEI37986	Zymop_1090	254	31.6% (vs BzaA)		aldehyde dehydrogenase
BA_AP_Sp_Ery	BzaA	Erythrobacter (Species, 3 taxa)	Bacteria		Proteobacteria	Alphaproteobacteria	Sphingomonadales	Erythrobacteraceae	Erythrobacter	Erythrobacter sp. SD-21	EDL49360	ED21_21809	403	45.4% (vs BzaA)	BzaA	aldehyde dehydrogenase
BA_AP_Rhos	BzaA	Rhodospirillales (Order, 15 taxa)	Bacteria		Proteobacteria	Alphaproteobacteria	Rhodospirillales			Acidiphilium cryptum JF-5	ABQ29502	Acry_0274	275	33.7% (vs BzaA)		succinate semialdehyde dehydrogenase
BA_AP_Cau	BzaA	Caulobacteraceae (Family, 9 taxa)	Bacteria		Proteobacteria	Alphaproteobacteria	Caulobacterales	Caulobacteraceae		Caulobacter sp. K31	ABZ69575	Caul_0438	488	54.2% (vs BzaA)	BzaA	aldehyde dehydrogenase
BA_BP_Bur	BzaA	Burkholderiales (Order, 66 taxa)	Bacteria		Proteobacteria	Betaproteobacteria	Burkholderiales			Ralstonia eutropha JMP134	AAZ60829	Reut_A1459	468	51.3% (vs BzaA)	BzaA	Aldehyde dehydrogenase (NAD+)
BA_BP_Nei	BzaA	Neisseriaceae (Family, 23 taxa)	Bacteria		Proteobacteria	Betaproteobacteria	Neisseriales	Neisseriaceae		Pseudogulbenkiania ferrooxidans 2002	EEG08593	FuraDRAFT_2101	304	36.4% (vs BzaA)		Aldehyde Dehydrogenase
BA_BP_Rho	BzaA	Rhodocyclaceae (Family, 6 taxa)	Bacteria		Proteobacteria	Betaproteobacteria	Rhodocyclales	Rhodocyclaceae		Aromatoleum aromaticum EbN1	CAI08943	ebA4954	435	47.4% (vs BzaA)	BzaA	Aldehyde dehydrogenase family protein
BA_BP_Nit	BzaA	Nitrosomonadaceae (Family, 5 taxa)	Bacteria		Proteobacteria	Betaproteobacteria	Nitrosomonadales	Nitrosomonadaceae		Nitrosomonas eutropha C91	ABI58972	Neut_0700	234	30.8% (vs BzaA)		aldehyde dehydrogenase
BA_BP_Met	BzaA	Methylophilales (Order, 5 taxa)	Bacteria		Proteobacteria	Betaproteobacteria	Methylophilales			Methylovorus glucosetrophus SIP3-4	ACT49361	Msip34_0112	384	44.1% (vs BzaA)	BzaA	aldehyde dehydrogenase
BA_DelEpsP	BzaA	deltaepsilon subdivisions (Class, 91 taxa)	Bacteria		Proteobacteria	Delta/Epsilon subdivision (no lank)				Desulfovibrio desulfuricans ND132	EGB14252	DND132_1039	305	39.4% (vs BzaA)		aldehyde dehydrogenase
BA_Firm_Baci	BzaA	Bacilli (Class, 201 taxa)	Bacteria		Firmicutes	Bacilli				Bacillus pumilus	KRU16515	AS142_11840	335	38.3% (vs BzaA)		betaine aldehyde dehydrogenase
BA_Firm_Clos	BzaA	Clostridia (Class, 174 taxa)	Bacteria		Firmicutes	Clostridia				Sulfobacillus acidophilus TPY	AEJ41144	TPY_2986	325	39.0% (vs BzaA)		gamma-aminobutyraldehyde dehydrogenase
BA_Actino	BzaA	Actinobacteria class (Class, 181 taxa)	Bacteria		Actinobacteria	Actinobacteria				Thermomonospora curvata DSM 43183	ACY98055	Tcur_2494	422	47.8% (vs BzaA)	BzaA	Betaine-aldehyde dehydrogenase
BA_Bacteroid	BzaA	BacteroidetesChlorobi group (Phylum, 145 taxa)	Bacteria		Bacteroidetes and Chlorobi (no rank)					unidentified eubacterium SCB49	EDM44314	SCB49_04775	362	40.0% (vs BzaA)	BzaA	aldehyde dehydrogenase
BA_Cyano	BzaA	Cyanobacteria (Phylum, 57 taxa)	Bacteria		Cyanobacteria					Lyngbya sp. PCC 8106	EAW38876	L8106_01137	325	39.3% (vs BzaA)		aldehyde dehydrogenase
BA_Deino	BzaA	Deinococci (Class, 15 taxa)	Bacteria		Deinococcus-Thermus	Deinococci				Marinithermus hydrothermalis DSM 14884	AEB12019	Marky_1279	315	40.0% (vs BzaA)		aldehyde dehydrogenase
BA_Chloro	BzaA	Chloroflexi (Phylum, 14 taxa)	Bacteria		Chloroflexi					Ktedonobacter racemifer DSM 44963	EFH82157	Krac_2942	318	37.5% (vs BzaA)		aldehyde dehydrogenase
BA_Euc_Meta	BzaA	Metazoa (Kingdom, 89 taxa)	Eukaryotes	Metazoa						Nasonia vitripennis	XP_001604192	LOC100120559	324	36.6% (vs BzaA)		aldehyde dehydrogenase, mitochondrial
BA_Euc_Fung	BzaA	Fungi (Kingdom, 85 taxa)	Eukaryotes	Fungi						Arthroderma otae CBS 113480	EEQ28274	MCYG_01162	411	44.3% (vs BzaA)	BzaA	aldehyde dehydrogenase
BA_Euc_Virid	BzaA	Viridiplantae (Kingdom, 23 taxa)	Eukaryotes	Viridiplantae						Selaginella moellendorffii	EFJ21291	SELMODRAFT_268124	302	37.6% (vs BzaA)		hypothetical protein
BA_Euc_Alveo	BzaA	Alveolata (Kingdom, 16 taxa)	Eukaryotes	Alveolata (no rank)						Perkinsus marinus ATCC 50983	XP_002784176	Pmar_PMAR003431	339	39.0% (vs BzaA)	BzaA	Succinate-semialdehyde dehydrogenase
BA_Arc_Eurya	BzaA	Euryarchaeota (Phylum, 79 taxa)	Archaea		Euryarchaeota					Methanocella arvoryzae MRE50	CAJ35595	RCIX91	332	39.6% (vs BzaA)		succinate-semialdehyde dehydrogenase
BA_Arc_Themo	BzaA	Thermoprotei (Class, 30 taxa)	Archaea		Crenarchaeota	Thermoprotei				Sulfolobus acidocaldarius DSM 639	AAY81006	Saci_1700	247	31.1% (vs BzaA)		aldehyde dehydrogenase
LV_GP_Entero	LigV	Enterobacteriaceae (Family, 83 taxa)	Bacteria		Proteobacteria	Gammaproteobacteria	Enterobacterales	Enterobacteriaceae		Enterobacter aerogenes KCTC 2190	AEG98929	EAE_20110	523	54.5% (vs LigV)	LigV/Vdh	NAD-dependent aldehyde dehydrogenase
LV_GP_Altero	LigV	Alteromonadales (Order, 41 taxa)	Bacteria		Proteobacteria	Gammaproteobacteria	Alteromonadales			Alteromonas naphthalenivorans	AEF05082	ambt_17915	498	49.6% (vs LigV)	LigV/Vdh	aldehyde dehydrogenase
LV_GP_Pseudo	LigV	Pseudomonadales (Order, 38 taxa)	Bacteria		Proteobacteria	Gammaproteobacteria	Pseudomonadales			Pseudomonas fluorescens F113	AEV62664	PSF113_2660	554	56.8% (vs LigV)	LigV/Vdh	aldehyde dehydrogenase
LV_GP_Vibrio	LigV	Vibrionales (Order, 29 taxa)	Bacteria		Proteobacteria	Gammaproteobacteria	Vibrionales			Vibrio sp. EJY3	AEX24729	VEJY3_21581	276	33.4% (vs LigV)		succinate semialdehyde dehydrogenase
LV_GP_Oceno	LigV	Oceanospirillales (Order, 13 taxa)	Bacteria		Proteobacteria	Gammaproteobacteria	Oceanospirillales			Halomonas elongata DSM 2581	CBV43816	HELO_3932	555	53.4% (vs LigV)	LigV/Vdh	aldehyde dehydrogenase family protein
LV_GP_Xantho	LigV	Xanthomonadaceae (Family, 10 taxa)	Bacteria		Proteobacteria	Gammaproteobacteria	Xanthomonadales	Xanthomonadaceae		Xanthomonas oryzae pv. oryzicola BLS256	AEQ98616	XOC_4559	295	33.6% (vs LigV)	LigV/Vdh	aldehyde dehydrogenase family protein
LV_AP_Rhizo	LigV	Rhizobiales (Order, 67 taxa)	Bacteria		Proteobacteria	Alphaproteobacteria	Rhizobiales			Methylobacterium sp. 4-46	ACA17864	M446_3479	672	72.0% (vs LigV)	LigV/Vdh	aldehyde dehydrogenase
LV_AP_Rhodo	LigV	Rhodobacterales (Order, 52 taxa)	Bacteria		Proteobacteria	Alphaproteobacteria	Rhodobacterales			Paracoccus sp. TRP	WP_010397469	PaTRP_010100013244	636	65.0% (vs LigV)	LigV/Vdh	aldehyde dehydrogenase
LV_AP_Ricke	LigV	Rickettsiales (Order, 23 taxa)	Bacteria		Proteobacteria	Alphaproteobacteria	Rickettsiales			Rickettsia akari str. Hartford	ABV74960	A1C_03370	230	28.5% (vs LigV)		succinate semialdehyde dehydrogenase
LV_AP_Sp_NA	LigV	Novosphingobium aromaticivorans (Species, 1)	Bacteria		Proteobacteria	Alphaproteobacteria	Sphingomonadales	Sphingomonadaceae	Novosphingobium	Novosphingobium aromaticivorans DSM 12444	ABD26108	Saro_1668	805	84.2% (vs LigV)	LigV/Vdh	vanillin dehydrogenase
LV_AP_Sp_SC	LigV	Sphingobium chlorophenolicum (Species, 1)	Bacteria		Proteobacteria	Alphaproteobacteria	Sphingomonadales	Sphingomonadaceae	Sphingobium	Sphingobium chlorophenolicum L-1	AEG51482	Sphch_3901	615	61.1% (vs LigV)	LigV/Vdh	Salicylaldehyde dehydrogenase
LV_AP_Sp_SJ	LigV	Sphingobium japonicum (Species, 1)	Bacteria		Proteobacteria	Alphaproteobacteria	Sphingomonadales	Sphingomonadaceae	Sphingobium	Sphingobium japonicum UT26S	BAI98456	SJA_C2-00930	259	32.7% (vs LigV)		succinate-semialdehyde dehydrogenase (NADP+)
LV_AP_Sp_SW	LigV	Sphingomonas wittichii (Species, 1)	Bacteria		Proteobacteria	Alphaproteobacteria	Sphingomonadales	Sphingomonadaceae	Sphingobium	Sphingomonas wittichii RW1	ABQ67449	Swit_1083	686	72.6% (vs LigV)	LigV/Vdh	aldehyde dehydrogenase
LV_AP_Sp_CB	LigV	Citromicrobium bathyomarinum (Species, 1)	Bacteria		Proteobacteria	Alphaproteobacteria	Sphingomonadales	Sphingomonadaceae	Citromicrobium	Citromicrobium sp. JL477	ALG60552	WG74_06670	320	38.0% (vs LigV)	LigV/Vdh	aldehyde dehydrogenase
LV_AP_Sp_ZM	LigV	Zymomonas mobilis (Species, 2)	Bacteria		Proteobacteria	Alphaproteobacteria	Sphingomonadales	Sphingomonadaceae	Zymomonas	Zymomonas mobilis subsp. pomaceae ATCC 29192	AEI37986	Zymop_1090	249	32.1% (vs LigV)		Succinate-semialdehyde dehydrogenase
LV_AP_Sp_Ery	LigV	Erythrobacter (Species, 3 taxa)	Bacteria		Proteobacteria	Alphaproteobacteria	Sphingomonadales	Erythrobacteraceae	Erythrobacter	Erythrobacter sp. SD-21	EDL49360	ED21_21809	230	30.2% (vs LigV)	BzaA	aldehyde dehydrogenase family protein
LV_AP_Rhos	LigV	Rhodospirillales (Order, 15 taxa)	Bacteria		Proteobacteria	Alphaproteobacteria	Rhodospirillales			Acetobacter pasteurianus IFO 3283-01	BAH98615	APA01_04640	254	32.1% (vs LigV)		aldehyde/betaine dehydrogenase
LV_AP_Cau	LigV	Caulobacteraceae (Family, 9 taxa)	Bacteria		Proteobacteria	Alphaproteobacteria	Caulobacterales	Caulobacteraceae		Caulobacter segnis ATCC 21756	ADG10805	Cseg_2346	746	75.8% (vs LigV)	LigV/Vdh	aldehyde dehydrogenase
LV_BP_Bur	LigV	Burkholderiales (Order, 66 taxa)	Bacteria		Proteobacteria	Betaproteobacteria	Burkholderiales			Burkholderia sp. CCGE1003	ADN60182	BC1003_4248	595	60.5% (vs LigV)	LigV/Vdh	aldehyde dehydrogenase
LV_BP_Nei	LigV	Neisseriaceae (Family, 23 taxa)	Bacteria		Proteobacteria	Betaproteobacteria	Neisseriales	Neisseriaceae		Pseudogulbenkiania ferrooxidans 2002	EEG07063	FuraDRAFT_3494	261	30.5% (vs LigV)		Aldehyde Dehydrogenase
LV_BP_Rho	LigV	Rhodocyclaceae (Family, 6 taxa)	Bacteria		Proteobacteria	Betaproteobacteria	Rhodocyclales	Rhodocyclaceae		Thauera sp. MZ1T	ACK54591	Tmz1t_1839	579	59.5% (vs LigV)	LigV/Vdh	betaine aldehyde dehydrogenase
LV_BP_Nit	LigV	Nitrosomonadaceae (Family, 5 taxa)	Bacteria		Proteobacteria	Betaproteobacteria	Nitrosomonadales	Nitrosomonadaceae		Nitrosomonas eutropha C91	ADZ27487	NAL212_2697	335	36.6% (vs LigV)	LigV/Vdh	Betaine-aldehyde dehydrogenase
LV_BP_Met	LigV	Methylophilales (Order, 5 taxa)	Bacteria		Proteobacteria	Betaproteobacteria	Methylophilales			Methylotenera versatilis 301	ADI29453	M301_1069	243	30.2% (vs LigV)		succinic semialdehyde dehydrogenase
LV_DelEpsP	LigV	deltaepsilon subdivisions (Class, 91 taxa)	Bacteria		Proteobacteria	Delta/Epsilon subdivision (no lank)				Geobacter lovleyi SZ	ACD94635	Glov_0912	375	39.5% (vs LigV)	LigV/Vdh	aldehyde dehydrogenase
LV_Firm_Baci	LigV	Bacilli (Class, 201 taxa)	Bacteria		Firmicutes	Bacilli				Geobacillus sp. Y4.1MC1	ADP73405	GY4MC1_0573	340	36.7% (vs LigV)	LigV/Vdh	aldehyde dehydrogenase
LV_Firm_Clos	LigV	Clostridia (Class, 174 taxa)	Bacteria		Firmicutes	Clostridia				Clostridium bolteae ATCC BAA-613	EDP16458	CLOBOL_03225	343	37.5% (vs LigV)	LigV/Vdh	hypothetical protein
LV_Actino	LigV	Actinobacteria class (Class, 181 taxa)	Bacteria		Actinobacteria	Actinobacteria				Micromonospora sp. ATCC 39149	EEP74825	MCAG_05152	468	48.4% (vs LigV)	LigV/Vdh	aldehyde dehydrogenase
LV_Bacteroid	LigV	BacteroidetesChlorobi group (Phylum, 145 taxa)	Bacteria		Bacteroidetes and Chlorobi (no rank)					Rhodothermus marinus DSM 4252	ACY48193	Rmar_1303	268	31.5% (vs LigV)		aldehyde dehydrogenase
LV_Cyano	LigV	Cyanobacteria (Phylum, 57 taxa)	Bacteria		Cyanobacteria					Cyanothece sp. ATCC 51142	ACB52577	cce_3229	266	32.9% (vs LigV)		aldehyde dehydrogenase
LV_Deino	LigV	Deinococci (Class, 15 taxa)	Bacteria		Deinococcus-Thermus	Deinococci				Thermus sp. CCB_US3_UF1	AEV16338	TCCBUS3UF1_12960	580	58.6% (vs LigV)	LigV/Vdh	aldehyde dehydrogenase
LV_Chloro	LigV	Chloroflexi (Phylum, 14 taxa)	Bacteria		Chloroflexi					Oscillochloris trichoides DG-6	EFO81201	OSCT_0935	265	33.6% (vs LigV)		aldehyde dehydrogenase
LV_Euc_Meta	LigV	Metazoa (Kingdom, 89 taxa)	Eukaryotes	Metazoa						Pediculus humanus corporis	XP_002424335	Phum_PHUM127180	273	31.8% (vs LigV)		Succinate semialdehyde dehydrogenase
LV_Euc_Fung	LigV	Fungi (Kingdom, 85 taxa)	Eukaryotes	Fungi						Colletotrichum graminicola M1.001	EFQ25459	GLRG_00603	391	41.6% (vs LigV)	LigV/Vdh	aldehyde dehydrogenase
LV_Euc_Virid	LigV	Viridiplantae (Kingdom, 23 taxa)	Eukaryotes	Viridiplantae						Physcomitrella patens	EDQ82380	PHYPADRAFT_113098	273	33.3% (vs LigV)		SSADH
LV_Euc_Alveo	LigV	Alveolata (Kingdom, 16 taxa)	Eukaryotes	Alveolata (no rank)						Perkinsus marinus ATCC 50983	XP_002784052	Pmar_PMAR003300	258	31.6% (vs LigV)		Betaine aldehyde dehydrogenase
LV_Arc_Eurya	LigV	Euryarchaeota (Phylum, 79 taxa)	Archaea		Euryarchaeota					Haladaptatus paucihalophilus DX253	EFW92248	ZOD2009_12245	293	33.7% (vs LigV)	LigV/Vdh	aldehyde dehydrogenase
LV_Arc_Themo	LigV	Thermoprotei (Class, 30 taxa)	Archaea		Crenarchaeota	Thermoprotei				Sulfolobus acidocaldarius DSM 639	AAY81163	Saci_1857	334	35.9% (vs LigV)	LigV/Vdh	aldehyde dehydrogenase

Table S6. Strains and plasmids used in this study

Strains or plasmids	Relevant characteristic(s)[a]	Source or reference
Strains		
Sphingobium sp.		
SYK-6	Wild type; Nal^r Sm^r	(66)
DLV	SYK-6 derivative; *ligV*::*kan*; Nal^r Sm^r Km^r	(67)
SME076	SYK-6 derivative; SLG_28320::*cat*; Nal^r Sm^r Cm^r	This study
SME077	SME076 derivative; SLG_28320::*cat ligV*::*kan*; Nal^r Sm^r Cm^r Km^r	This study
Escherichia coli		
JM109	*recA1 supE44 endA1 hsdR17*(r_K^- m_K^+) *gyrA96 relA1 thi-1* Δ(*lac-proAB*) F′[*traD36 proAB*$^+$ *lacI*^{q} *lacZ*ΔM15]	(68)
BL21(DE3)	F$^-$ *ompT hsdS$_B$*(r_B^- m_B^-) *gal dcm* (DE3); T7 RNA polymerase gene under the control of the *lacUV* promoter	(69)
Plasmids		
pBluescript II KS(+)	Cloning vector; Ap^r	(70)
pT7Blue	Cloning vector; Ap^r	Novagen
pUC19	Cloning vector; Ap^r	(68)
pET21a(+)	Expression vector, T7 promoter, Ap^r	Novagen
pET-16b	Expression vector, T7 promoter, Ap^r	Novagen
pK18*mobsacB*	*oriT sacB* Km^r	(71)
pIK03	Kanamycin cassette; Ap^r Km^r	(72)
pJB866	RK2 broad-host-range expression vector; Tc^r P_m *xylS*	(73)
pLVH	pET21a(+) with a 1.9-kb NdeI-XhoI fragment carrying *ligV*	(67)
pIK34D	pK19*mobsacB* with a *ligV*::*kan* fragment	(67)
pT7Cm	pT7Blue with a 1.0-kb PCR amplified fragment carrying *cat*	(74)
pTLV530	pT7Blue with a 1.5-kb PCR amplified fragment carrying *ligV*	This study
pKS0727	KS(+) with a 1.5-kb PCR amplified fragment carrying SLG_07270	This study
pKS0761	KS(+) with a 1.5-kb PCR amplified fragment carrying SLG_07610	This study
pKS0779	KS(+) with a 1.5-kb PCR amplified fragment carrying SLG_07790	This study
pKS0940	KS(+) with a 1.5-kb PCR amplified fragment carrying SLG_09400	This study
pKS0951	KS(+) with a 1.5-kb PCR amplified fragment carrying SLG_09510	This study
pKS1202	KS(+) with a 1.5-kb PCR amplified fragment carrying SLG_12020	This study
pKS1219	KS(+) with a 1.5-kb PCR amplified fragment carrying SLG_12190	This study
pKS1280	KS(+) with a 1.5-kb PCR amplified fragment carrying SLG_12800	This study
pKS1821	KS(+) with a 1.5-kb PCR amplified fragment carrying SLG_18210	This study
pKS2040	KS(+) with a 1.5-kb PCR amplified fragment carrying SLG_20400	This study
pKS2721	KS(+) with a 1.5-kb PCR amplified fragment carrying SLG_27210	This study
pKS2791	KS(+) with a 1.5-kb PCR amplified fragment carrying *bzaA*	This study
pKS2792	KS(+) with a 1.5-kb PCR amplified fragment carrying *bzaB*	This study
pKS2815	KS(+) with a 1.5-kb PCR amplified fragment carrying SLG_28150	This study
pKS2832	KS(+) with a 1.5-kb PCR amplified fragment carrying SLG_28320	This study
pKSp0068	KS(+) with a 1.5-kb PCR amplified fragment carrying SLG_p_00680	This study
pK18-2832	pK18*mobsacB* with a 1.5-kb HindIII-XbaI fragment carrying SLG_28320	This study
pK18-2832Cm	pK18-2632 with a 1.0-kb SmaI fragment carrying *cat* from pT7Cm into AleI site of SLG_28320	This study
pT09920	pT7Blue with a 1.5-kb PCR amplified fragment carrying SLG_09920	This study
pT11410	pT7Blue with a 1.5-kb PCR amplified fragment carrying SLG_11410	This study
pT31150	pT7Blue with a 1.5-kb PCR amplified fragment carrying SLG_31150	This study
pT32240	pT7Blue with a 1.5-kb PCR amplified fragment carrying SLG_32240	This study
pT34940T	pT7Blue with a 1.2-kb PCR amplified fragment carrying 5' region of SLG_34940	This study
pT34940B	pT7Blue with a 0.3-kb PCR amplified fragment carrying 3' region of SLG_34940	This study
pT38120T	pT7Blue with a 0.2-kb PCR amplified fragment carrying 5' region of SLG_38120	This study
pT38120B	pT7Blue with a 1.3-kb PCR amplified fragment carrying 3' region of SLG_38120	This study
pT21-0727	pET-21a(+) with a 1.5-kb NdeI-BamHI fragment of pKS0727 carrying SLG_07270	This study
pT21-0761	pET-21a(+) with a 1.5-kb NdeI-BamHI fragment of pKS0761 carrying SLG_07610	This study
pT21-0779	pET-21a(+) with a 1.5-kb NdeI-BamHI fragment of pKS0779 carrying SLG_07790	This study
pT21-0940	pET-21a(+) with a 1.5-kb NdeI-BamHI fragment of pKS0940 carrying SLG_09400	This study
pT21-0951	pET-21a(+) with a 1.5-kb NdeI-BamHI fragment of pKS0951 carrying SLG_09510	This study
pT21-0992	pET-21a(+) with a 1.5-kb NdeI-BamHI fragment of pT09920 carrying SLG_09920	This study
pT21-1141	pET-21a(+) with a 1.5-kb NdeI-BamHI fragment of pT11410 carrying SLG_11410	This study

pT21-1202	pET-21a(+) with a 1.5-kb NdeI-BamHI fragment of pKS1202 carrying SLG_12020	This study
pT21-1219	pET-21a(+) with a 1.5-kb NdeI-BamHI fragment of pKS1219 carrying SLG_12190	This study
pT21-1280	pET-21a(+) with a 1.5-kb NdeI-BamHI fragment of pKS1280 carrying SLG_12800	This study
pT21-1821	pET-21a(+) with a 1.5-kb NdeI-BamHI fragment of pKS1821 carrying SLG_18210	This study
pT21-2040	pET-21a(+) with a 1.5-kb NdeI-BamHI fragment of pKS2040 carrying SLG_20400	This study
pT21-2721	pET-21a(+) with a 1.5-kb NdeI-BamHI fragment of pKS2792 carrying SLG_27210	This study
pT21-2791	pET-21a(+) with a 1.5-kb NdeI-BamHI fragment of pKS2791 carrying *bzaA*	This study
pT21-2792	pET-21a(+) with a 1.5-kb NdeI-BamHI fragment of pKS2792 carrying *bzaB*	This study
pT21-2815	pET-21a(+) with a 1.5-kb NdeI-BamHI fragment of pKS2815 carrying SLG_28150	This study
pT21-2832	pET-21a(+) with a 1.5-kb NdeI-BamHI fragment of pKS2832 carrying SLG_28320	This study
pT21-3115	pET-21a(+) with a 1.5-kb NdeI-BamHI fragment of pT31150 carrying SLG_31150	This study
pT21-3224	pET-21a(+) with a 1.5-kb NdeI-BamHI fragment of pT32240 carrying SLG_32240	This study
pT21-3494B	pET-21a(+) with a 0.3-kb NdeI-BamHI fragment of pT34940B carrying 3' region of SLG_34940	This study
pT21-3494	pT21-3494B with a 1.2-kb NdeI fragment of pT34940T carrying 5' region of SLG_34940	This study
pT21-3812T	pET-21a(+) with a 0.2-kb NdeI-SalI fragment of pT38120T carrying 5' region of SLG_38120	This study
pT21-3812	pT21-3812T with a 1.3-kb SalI-BamHI fragment of pT38120B carrying 3' region of SLG_38120	This study
pT21-p0068	pET-21a(+) with a 1.5-kb NdeI-BamHI fragment of pKSp0068 carrying SLG_p_00680	This study
pT16-ligV	pET-16b with a 1.9-kb NdeI-XhoI fragment of pLVH carrying *ligV*	This study
pT16-desV	pET-16b with a 1.5-kb NdeI-BamHI fragment of pKS2832 carrying SLG_28320	This study
pJB28320	pJB866 with a 1.5-kb HindIII-XbaI fragment carrying SLG_28320	This study

[a]Abbreviations: Nal^r, Sm^r, Km^r, Tc^r, and Ap^r, resistance to nalidixic acid, streptomycin, kanamycin, tetracycline, and ampicillin, respectively.

Table S7. Primer sequences used in this study

Purposes	Target genes	Primers	Sequence (5′ to 3′)[a]
Construction of plasmids	SLG_07060	LigVNdeIF	TCCCCGCATATGGACTCAGC
		LigVhighNcoI4	CAGTCTCGCTCGCCTTCAGG
	SLG_07270	0415_V3-10_Nde_F	GAGCATATGGCCCAGCCAG
		0415_V3-10_Bam_R	AGGATCCGTGTGTCATCCGGTCAG
	SLG_07610	0453_V2-7_Nde_F	CATATGAACGATATCCAGAC
		0453_V2-7_Bam_R	AGGATCCAGCCCGTCTATCGGGAC
	SLG_07790	0471_V2-6_Nde_F	AAGCATATGAGCGAGGTGG
		0471_V2-6_Bam_R	AGGATCCGACAGAGCGGTACTGTGGTG
	SLG_09400	0644_E-19_Nde_F	CATATGTCTGAAGGCGAT
		0644_E-19_Bam_R	AGGATCCTCAGTTCCCTCCGTCCTTTA
	SLG_09510	0655_B-14_Nde_F	CATATGAACGCGCCTTATAATC
		0655_B-14_Bam_R	AGGATCCGACCGGATCAGGACTTGC
	SLG_09920	09920_Nde	AGATCATCCATATGCATGAGCTCGTTTCC
		09920_1020R	CCGTGCTTGGCCCCTATAATTTCC
		09920_Bam	ACCGGATCCGAAGGGCGAGCCTTGC
	SLG_11410	11410_Nde	AGGACCCACATATGGCAGACGCCG
		11410_Bam	TGGGGATCCGGTCGTTCTACGCAGAGG
	SLG_12020	0062_V3-11_Nde_F	GGACATATGCAATATAGCTACCTC
		0062_V3-11_Bam_R	AGGATCCTTATCCCGGCATGGAAAAG
	SLG_12190	0081_A-2_Nde_F	CATATGGATCAAAGTCAG
		0081_A-2_Bam_R	AGGATCCTGACAGGAAATCATGCCTCA
	SLG_12800	0145_A-3_Nde_F	CATATGACCGCCTATCCG
		0145_A-3_Bam_R	AGGATCCGTTTCCTGAATGGAAAGCC
	SLG_18210	0045_E-18_Xho_R	GACCTCGAGCGGGAAAGAATGCCTTCAG
		0045_E-18_Nde_F	CATATGACCGGTGCAATTCT
	SLG_20400	0286_D-17_Nde_F	AGGCATATGACCAGCTCCA
		0286_D-17_Bam_R	AGGATCCCGGTCTAACGAATCAGG
	SLG_27210	0098_C-15_Nde_F	CATATGGCCTCCATCGTTCAG
		0098_C-15_Bam_R	AGGATCCGCGTTCAGAAGAAGCCGAG
	SLG_27910	0172_V2-5_Nde_F	AACCATATGGACCATGAAT
		0172_V2-5_Bam_R	AGGATCCGCCCAGCTCTGAAAAAA
	SLG_27920	0173_V3-9_Nde_F	TGGCATATGACCCCTGACG
		0173_V3-9_Bam_R	AGGATCCGCTCGAGGACCGCTGAC
	SLG_28150	0002_E-20_Nde_F	CATATGACCGAAACCGCC
		0002_E-20_Bam_R	AGGATCCAAGACAGGGCTCATTTCTGG
	SLG_28320	0020_V3-8_Nde_F	CGCCATATGGATCAGTTCA
		0020_V3-8_Bam_R	AGGATCCATCCGTCGTCGGCTCAG
	SLG_31150	31150_Nde	TGGCGACGCATATGAGCCTGAACAGG
		31150_Bam	GTTGGATCCTTATCGTTTTCCCTGTCG
	SLG_32240	32240_Nde	ATAGCCCGCATATGTCGCAACCC
		32240_Bam	GAAGGATCCGGCCCGGCTCTGC
	SLG_34940	34940_Nde	GGGAGGACCATATGTATACTGACTCGC
		34940_Bam	CCGGATCCTCGCTCGAATATATCG
	SLG_38120	38120_Nde	GCCGCAGCATATGAGCGAGCCC
		38120_Xho	GGCCCTCGAGCTCGACATCAGCG
	SLG_p_00680	0130_C-16_Nde_F	CATATGTTCGAGAAGGCG
		0130_C-16_Bam_R	AGGATCCGTTTCTCCTTTTGAGCGAG
RT-PCR analyses	SLG_07060	002_0390_F2	CGTCATTCTCGGCATCG
		002_0390_R2	CAGTTCGATCGCATGCTC
	SLG_07270	002_0415_F2	GTTCGACAATATCCGGAAGG
		002_0415_R2	CGATGAAATAGCCCTGGTTG
	SLG_07610	002_0453_F2	GCTGACGATGCTCGATCTCT
		002_0453_R2	GTGACGATCGTGGGATCAAT
	SLG_07790	002_0471_F2	CACATGAAGCAGCTCTCCG
		002_0471_R2	GTCGGCTCGATGAAGTATCC
	SLG_09400	002_0644_F2	CACCCAGTTCGTGCCTTATC
		002_0644_R2	GATCGCTTCCTCGACCGT
	SLG_09510	002_0655_F2	GGCCAGTTGCGCTACAAT
		002_0655_R2	TGGACGAAATAGCCTTCCTC

	SLG_09920	002_0698_F2	CGTGCTTGGCCCCTATAAT
		002_0698_R2	GGCATCGAAATCCGTCTC
	SLG_11410	003_0029_F2	CATCCGCTACAATGCCG
		003_0029_R2	GATGGTCGGGTTGACAAAAT
	SLG_12020	004_0062_F2	GCTTCGAGGAGCAGGAATC
		004_0062_R2	GTCATGTCGTTGGTCACGTT
	SLG_12190	004_0081_F2	GAGGTCTATGCCGAGGAAGC
		004_0081_R2	GACATCGACGACCAGCGT
	SLG_12800	004_0145_F2	GCTCGTGCGTCCGGC
		004_0145_R2	GGCTCCTCGTTCATGATCTC
	SLG_18210	009_0045_F2	GCGCCACGATCTGACAT
		009_0045_R2	CGCACCAGAAGCGAAGTC
	SLG_20400	009_0286_F2	CCTGGAACTTCCCGCTC
		009_0286_R2	CTGCAGGATCTTCATCTGCG
	SLG_27210	016_0098_F2	GATCATTTCCGCTATTTCGC
		016_0098_R2	ACCGTCGGCTGGACATAGTA
	SLG_27910	016_0172_F2	CAACACGGTCATCGAACATC
		016_0172_R2	CATATTTGAGCACCGGCAG
	SLG_27920	016_0173_F2	AGAGCTATCCCTTCGTCGAG
		016_0173_R2	TATCGACATTCGCGAACAGG
	SLG_28150	017_0002_F2	GCATCGAGGCGCATTC
		017_0002_R2	CGCACCACGGAGAGGAC
	SLG_28320	017_0020_F2	CGGGCCTGTTCAAATTCTAT
		017_0020_R2	GGTCGGTTCGACGAAGAAG
	SLG_31150	018_0085_F2	CACTTATGGCGATGTGATCC
		018_0085_R2	ATGTCTCCTCGACGCACAG
	SLG_32240	018_0197_F2	ATCGCACCGCATGTGTTT
		018_0197_R2	CCGTAGCGGCTGTTGTTC
	SLG_34940	020_0161_F2	GCTTATGGTCGGGTCATTCC
		020_0161_R2	GAACGGCTCCTCGTTCATC
	SLG_38120	020_0505_F2	CTACGCTATAATGCCGGCTG
		020_0505_R2	CATATGCGGTTCGGCG
	SLG_p_00680	001_0130_F2	CATTTCCGCTATTTCGCC
		001_0130_R2	GTTCTGACCCACGAACACG
qRT-PCR analyses	SLG_07060	LigV_0390_qRT-F	AGATCGGCGCCACCAA
		LigV_0390_qRT-R	GCCGCCAGTCCGAGATT
	SLG_07270	10_0415_qRT-F	GGCGAACGACAGCGTCTAC
		10_0415_qRT-R	GCTTGCGGGTCCTTGGT
	SLG_07610	7_0453_qRT-F	CGCCTTCGCCAATTGC
		7_0453_qRT-R	CATAATGAGAGGTGTGGACGAAGA
	SLG_07790	6_0471_qRT-F	GCTCGCCATGCACAAGGT
		6_0471_qRT-R	GCTTCAGGATCAGCGTGTTG
	SLG_27910	5_0172_qRT-F	GGCGGGCAAGGTCTATCAG
		5_0172_qRT-R	CTTGATCGCCACGCAGATCT
	SLG_28320	8_0020_qRT-F	CGCCAAGGCCAATGACA
		8_0020_qRT-R	CGGCGTCCTGCGTGTAG
	16S rRNA gene	SYK-6-16S-F	GCGCAGAACCTTACCAACGT
		SYK-6-16S-R	AGCCATGCAGCACCTGTCA

[a]Nucleotides with underlines indicate a restriction enzyme site of NdeI, BamHI, and XhoI.

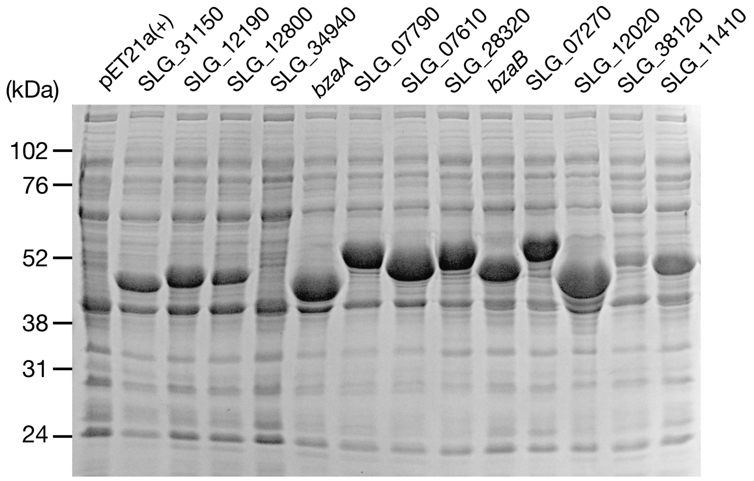

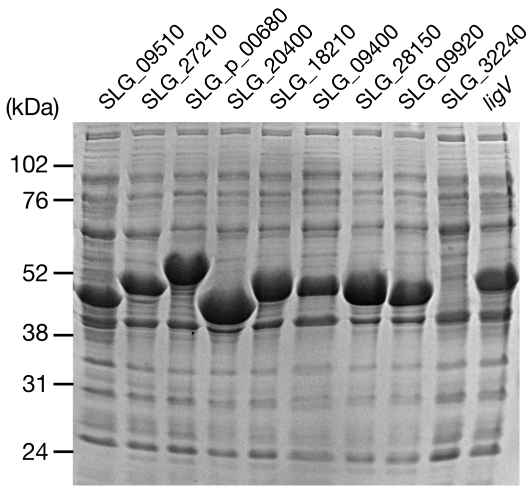

Fig. S1. Expression of ALDH genes of *Sphingobium* sp. SYK-6 in *E. coli*. Cell extracts of *E. coli* BL21(DE3) which harbor pET21a(+) carrying SYK-6 ALDH genes were separated by SDS-12% polyacrylamide gels and stained with Coomassie brilliant blue.

SN conversion

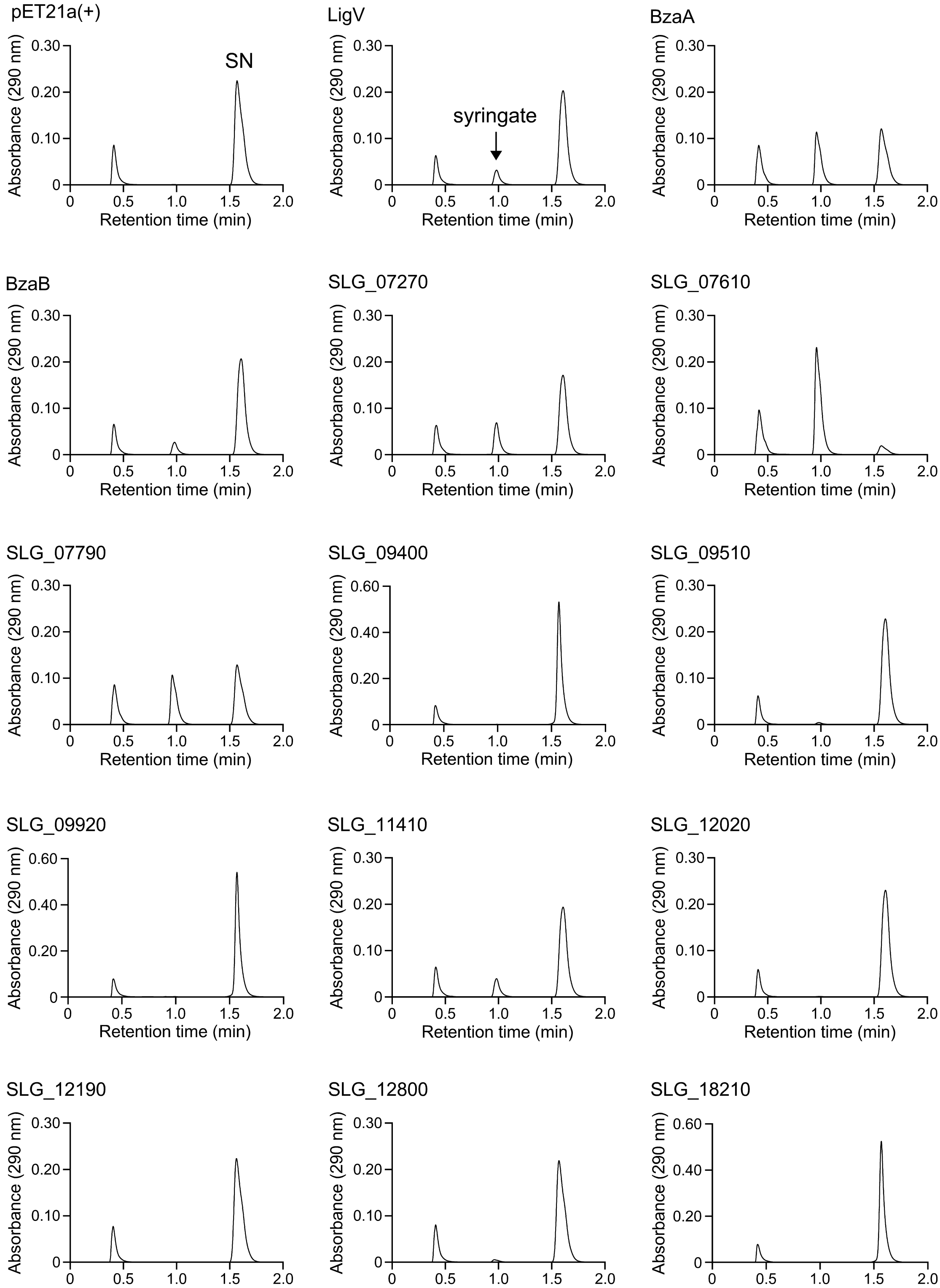

Fig. S2. HPLC analysis of SN and VN conversions by extracts of *E. coli* cells carrying SYK-6 ALDH genes. After incubation of cell extracts with 100 μM SN or VN in the presence of 500 μM NAD^+ for 5.0 min, portions of the mixture were analyzed using HPLC. SN and VN were detected at 290 nm. The retention times of SN, VN, syringate, and vanillate were 1.57 min, 1.47 min, 0.97 min, and 0.97 min, respectively.

SN conversion

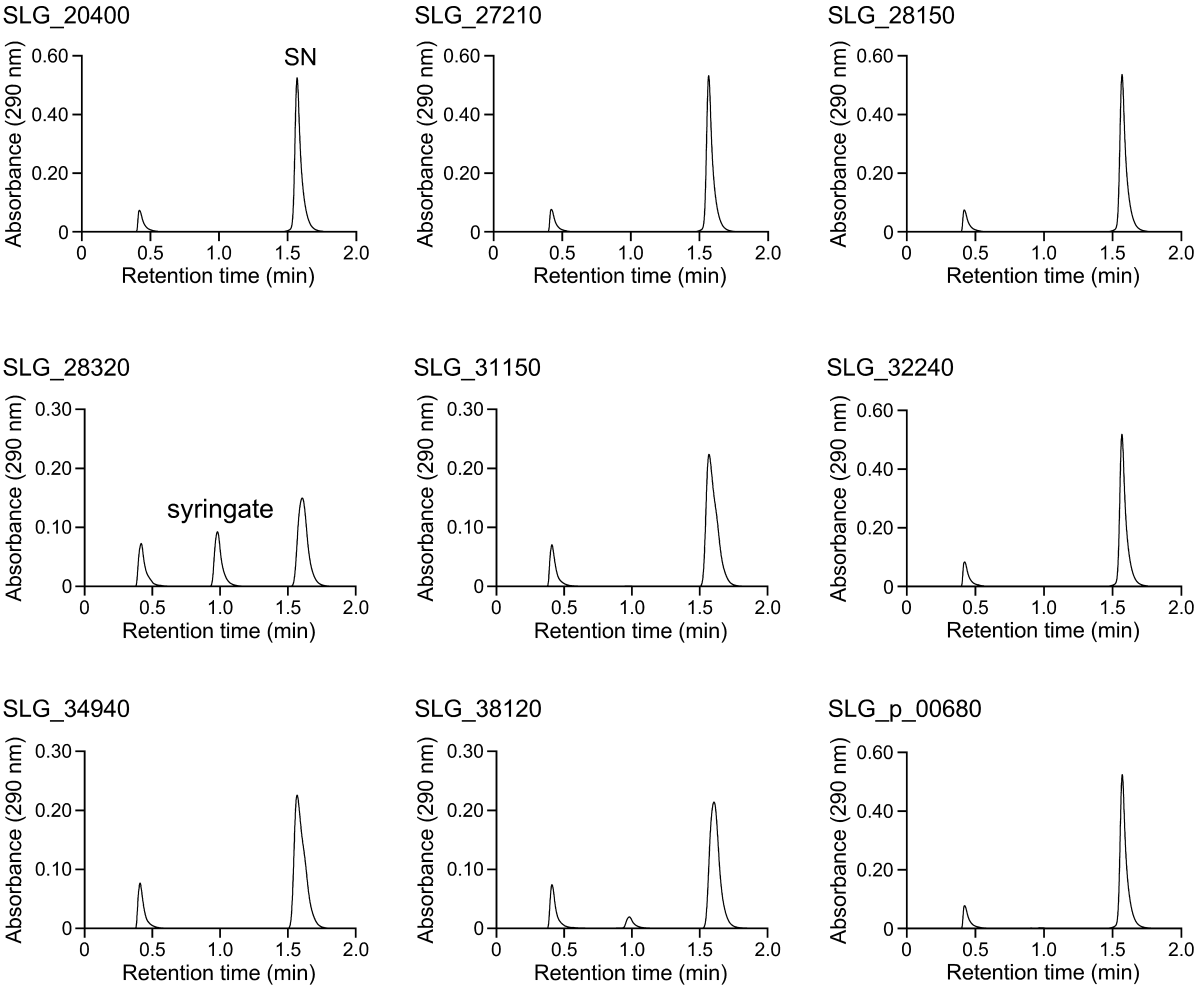

Fig. S2—*Continued.*

VN conversion

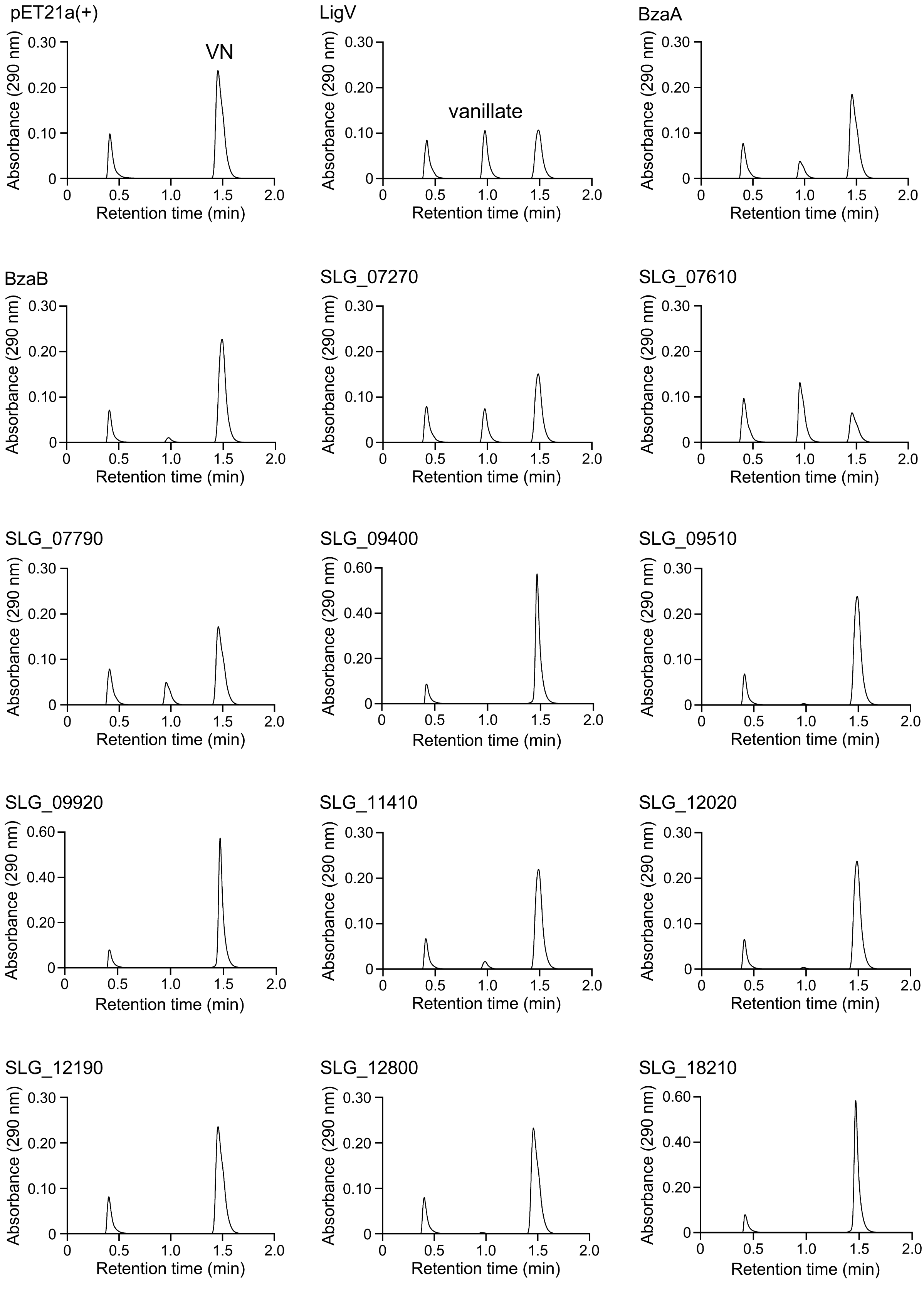

Fig. S2—*Continued.*

VN conversion

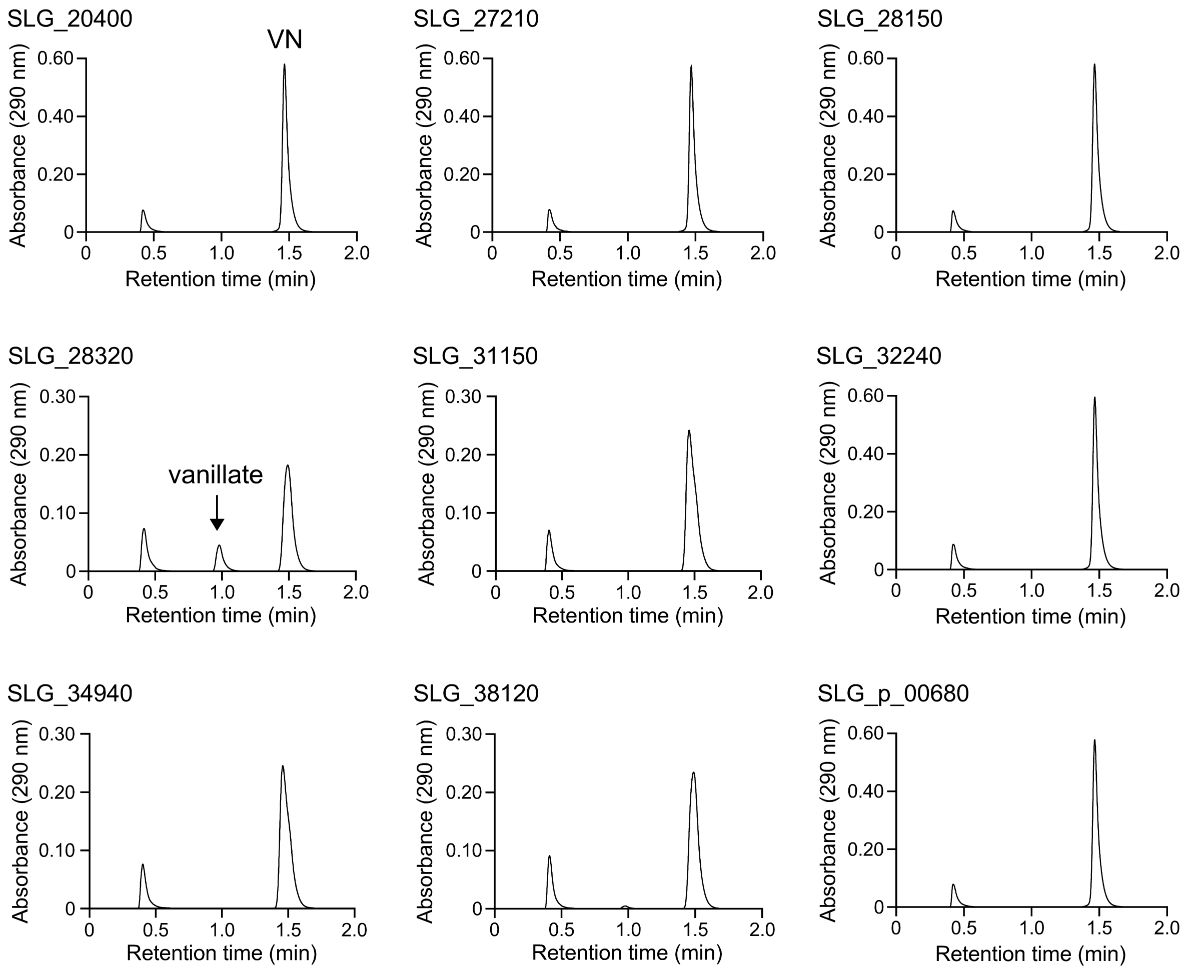

Fig. S2—*Continued.*

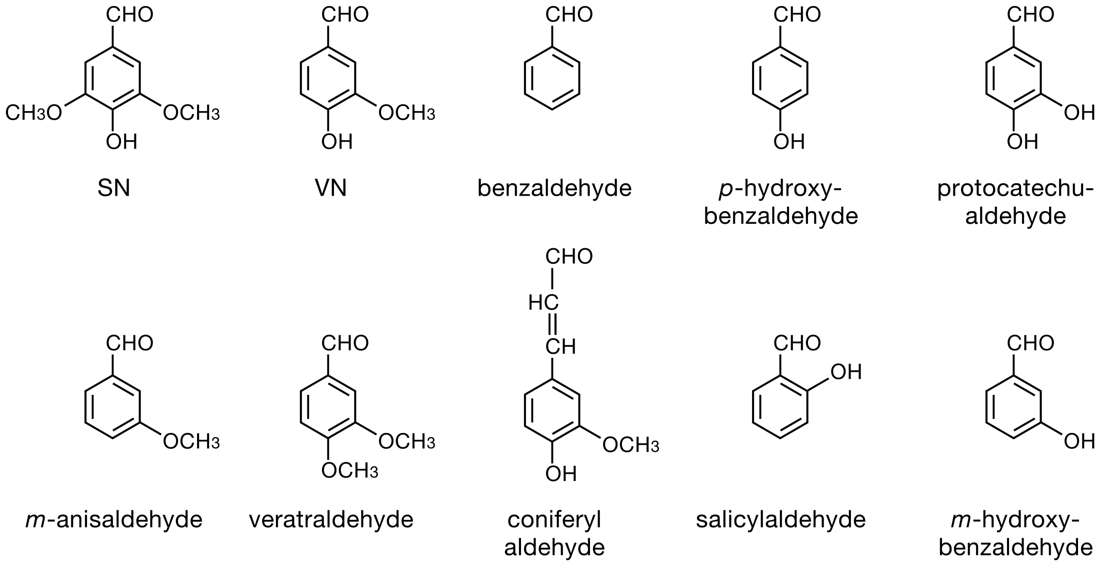

Fig. S3. Chemical structures of benzaldehyde derivatives used to examine the substrate range of ALDHs.

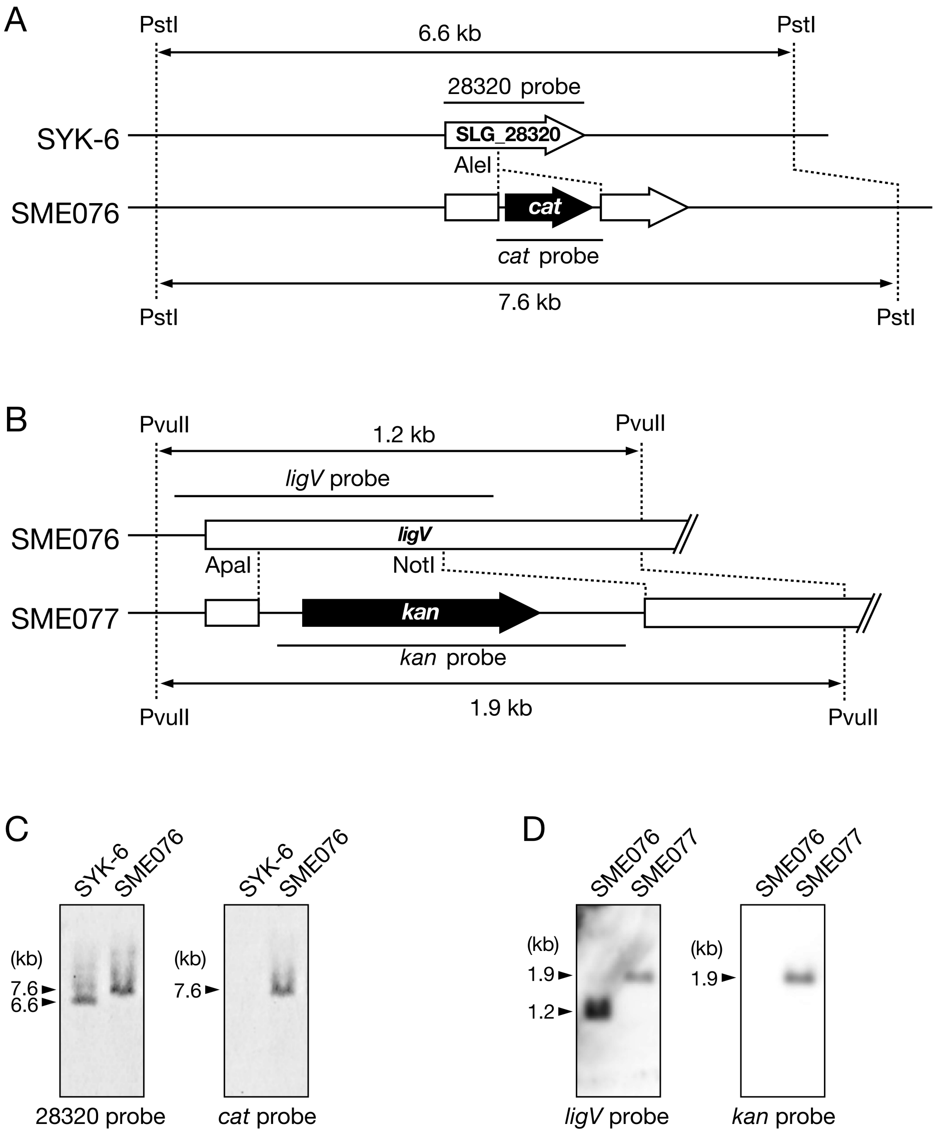

Fig. S4. Disruption of SLG_28320 and *ligV* in *Sphingobium* sp. SYK-6. Schematic representations of the disruption of SLG_28320 in SYK-6 (A) and the disruption of *ligV* in SLG_28320 mutant, SME076 (B) by the insertion of a chloramphenicol resistance gene (*cat*) and a kanamycin resistance gene (*kan*), respectively. (C) Southern hybridization analysis of SME076. Total DNA of SYK-6 and SME076 digested with PstI were hybridized with a 28320 probe and a *cat* probe. The positions of the probes are shown in panel A. (D) Southern hybridization analysis of SLG_28320 *ligV* double mutant, SME077. Total DNA of SME076 and SME077 digested with PvuII were hybridized with a *ligV* probe and a *kan* probe. The positions of the probes are shown in panel B.

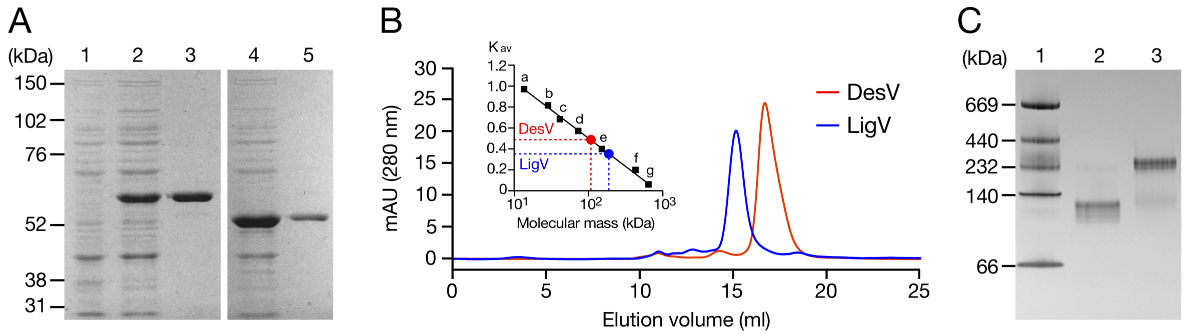

Fig. S5. Purification and molecular mass determination of DesV and LigV. (A) SDS-PAGE of DesV and LigV. Lanes: 1, cell extracts of *E. coli* BL21(DE3) harboring pET-16b (10 µg protein); 2, cell extracts of *E. coli* BL21(DE3) harboring pT16-desV (10 µg protein); 3, Purified DesV (2 µg protein); 4, cell extracts of *E. coli* BL21(DE3) harboring pT16-ligV; 5, Purified LigV (2 µg protein). (B) Size exclusion chromatography profiles of purified DesV (red) and LigV (blue). Molecular masses of standard proteins are as follows: (a) thyroglobulin (669 kDa); (b) ferritin (440 kDa); (c) aldolase (158 kDa); (d) conalbumin (75 kDa); (e) ovalbumin (43 kDa); (f) carbonic anhydrase (29 kDa); (g) ribonuclease A (13.7 kDa). (C) Native-PAGE of DesV and LigV. Purified proteins were separated on a 5–20% polyacrylamide gradient gel. Lanes: 1, molecular mass markers; 2, Purified DesV (4 µg protein); 3, Purified LigV (4 µg protein).

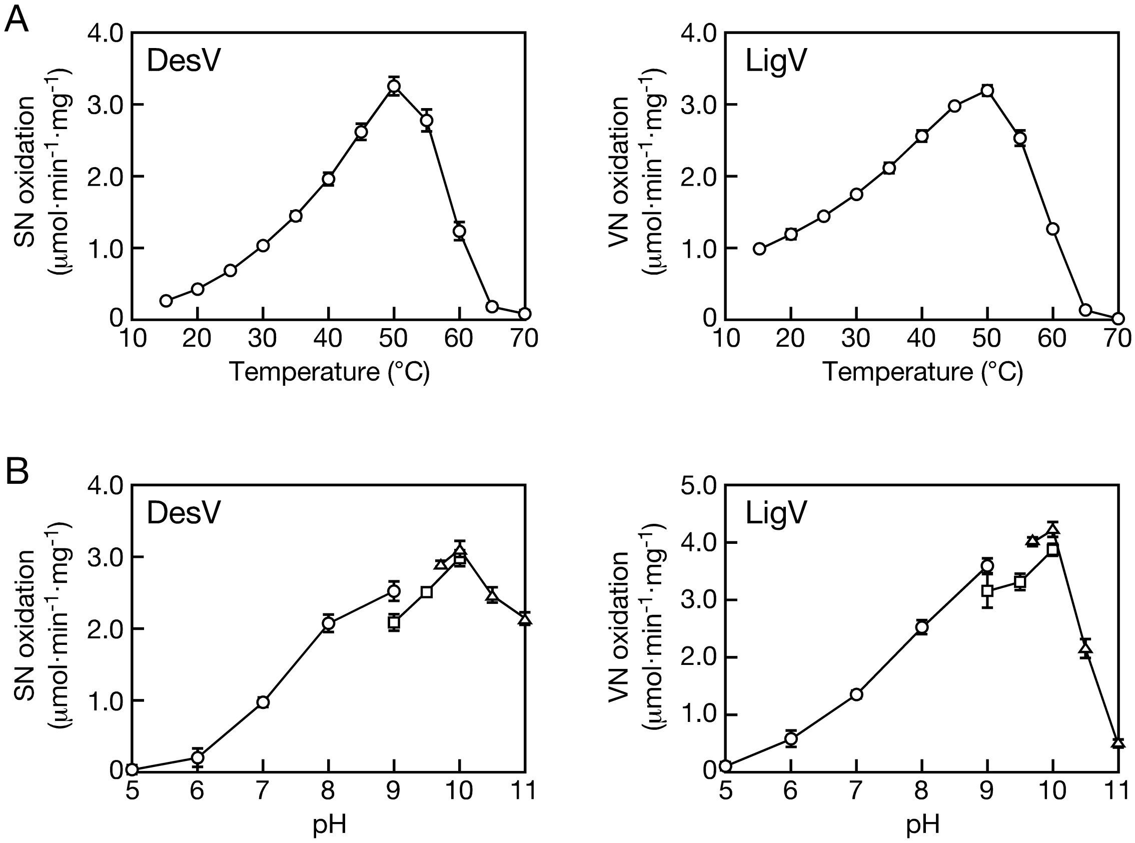

Fig. S6. Optimal temperature (A) and optimum pH (B) for DesV and LigV. (A) Oxidation activities of DesV for SN and LigV for VN were determined using 100 mM KH_2PO_4-K_2HPO_4 buffer (pH 7.0) at 15 to 70°C. (B) Oxidation activities of DesV for SN and LigV for VN were determined using 50 mM GTA buffer (pH 5.0 to 9.0; circles), 50 mM *N*-cyclohexyl-2-aminoethanesulfonate (pH 9.0 to 10.0; squares), and 50 mM *N*-cyclohexyl-3-aminopropanesulfonate (pH 9.7 to 11.0; triangles) at 30°C. The data are mean ± standard deviation of three independent experiments.

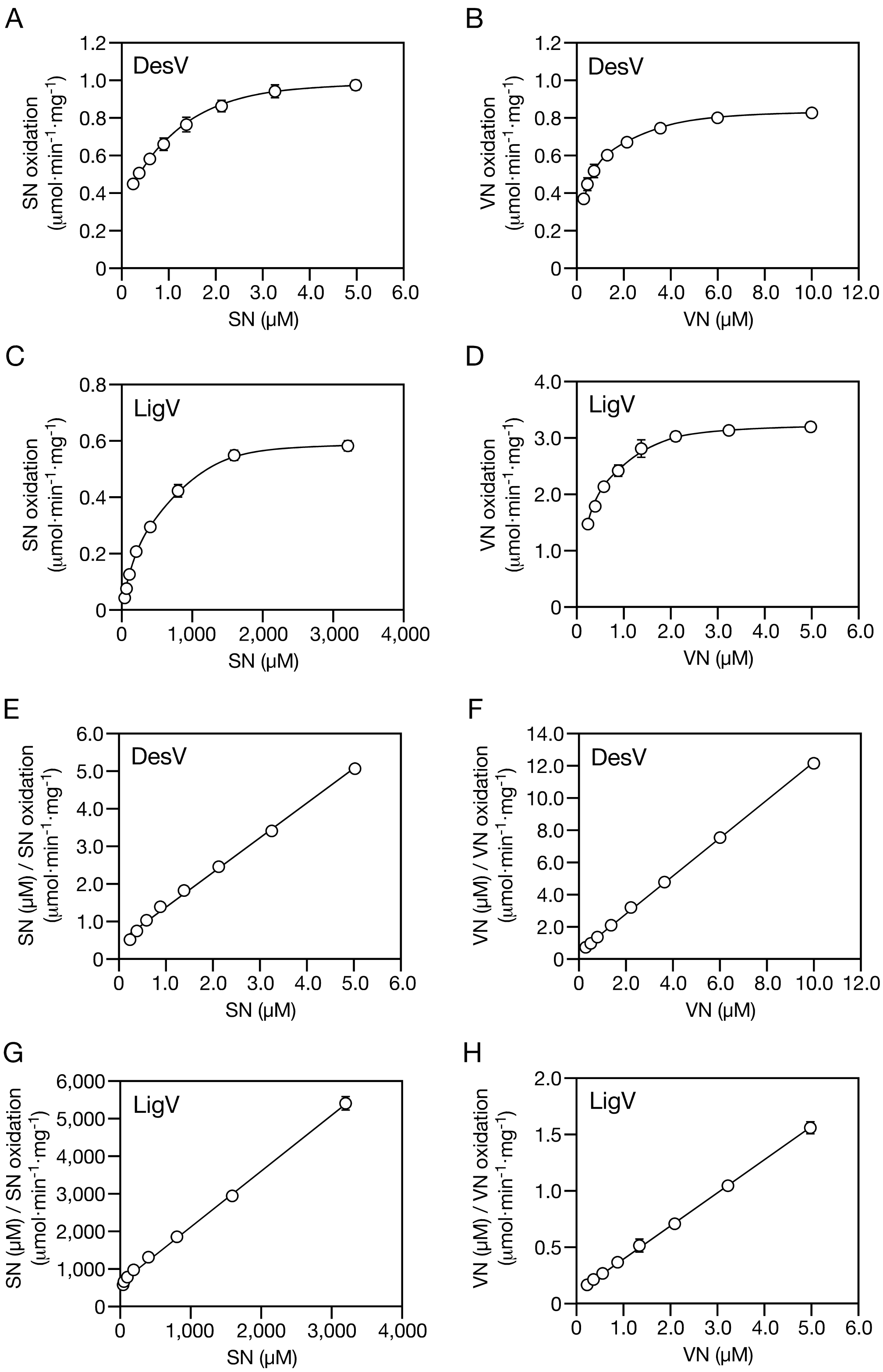

Fig. S7. Kinetic analysis of DesV and LigV. (A-D) Oxidation activities of SN and VN by DesV (A and B) and LigV (C and D) measured at various concentrations of the substrates. (E-H) Hanes-Woolf plots to determine the kinetic parameters of DesV (E and F) and LigV (G and H) for the oxidation of SN and VN. The data are mean ± standard deviation of three independent experiments.

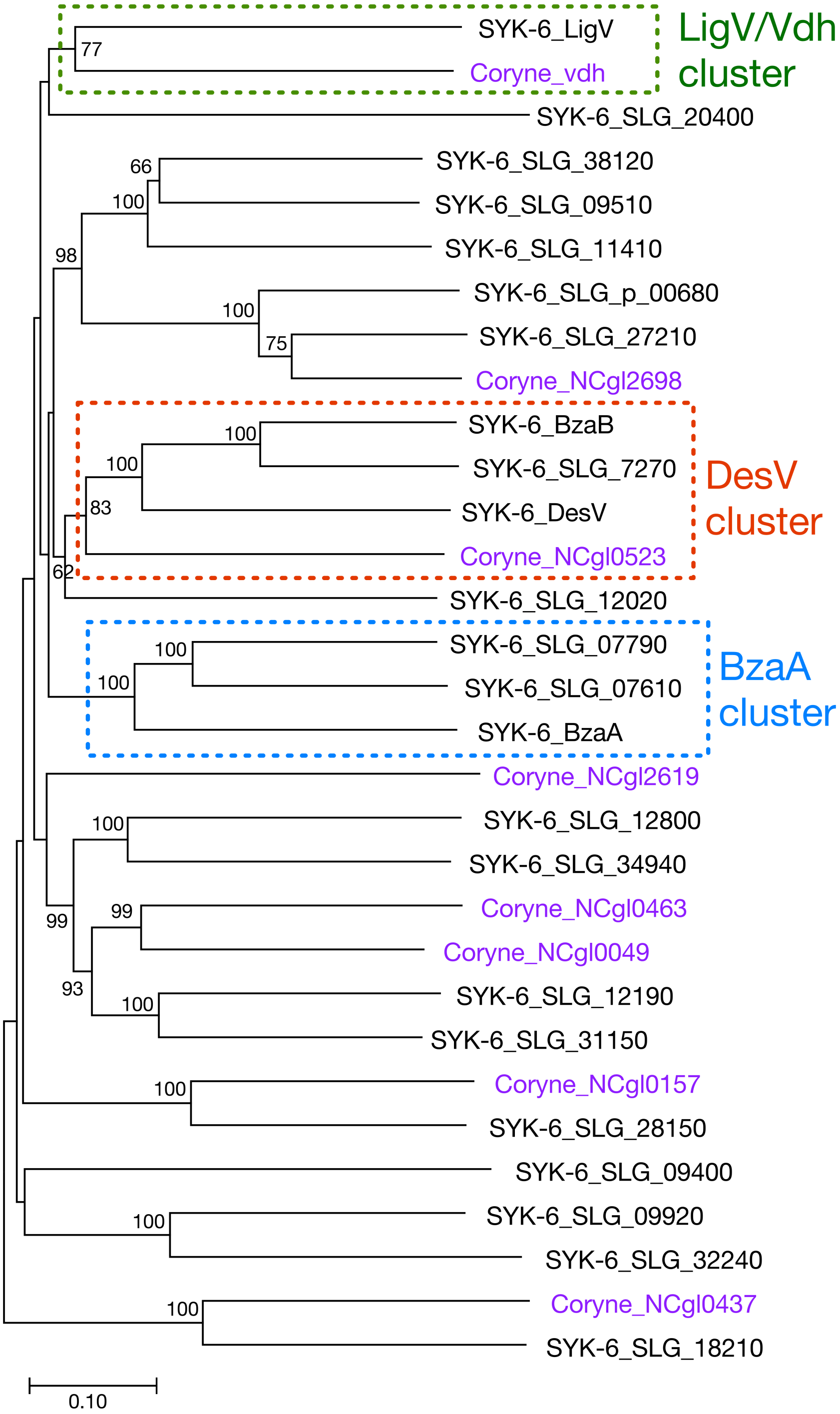

Fig. S8. Phylogenetic tree of putative ALDHs from *Corynebacterium glutamicum* ATCC 13032 and SYK-6 ALDHs. Putative ALDH genes of *C. glutamicum* ATCC 13032 were searched using the BLASTP program. The tree was constructed using the neighbor-joining algorithm with the Clustal W and MEGA 7 programs employing 1000 bootstrap replicates. Bootstrap values are indicated at the nodes, and the scale corresponds to an evolutionary distance of 0.1 amino acid substitutions per position. Putative ALDHs and Vdh from *C. glutamicum* ATCC 13032 are shown in purple.

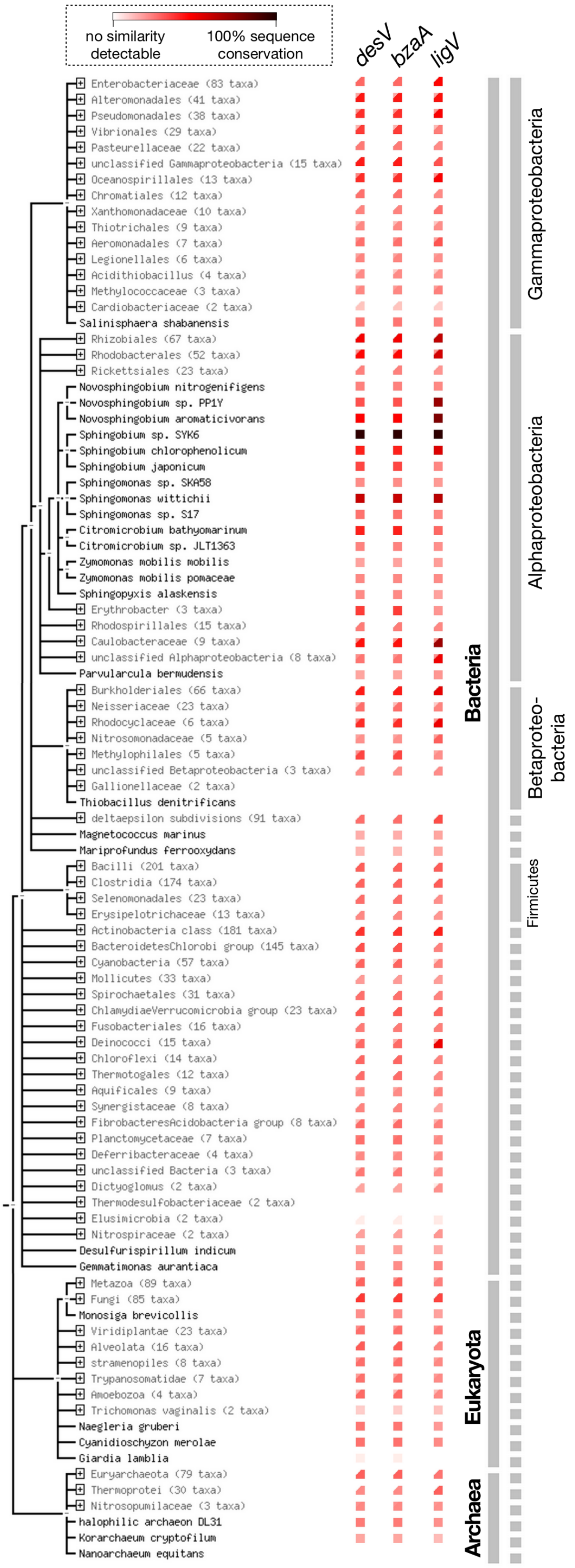

Fig. S9. Phylogenetic co-occurrence pattern of *desV*, *bzaA*, and *ligV*. The presence of homologous genes of *desV*, *bzaA*, and *ligV* were surveyed using the STRING database (http://string-db.org/).

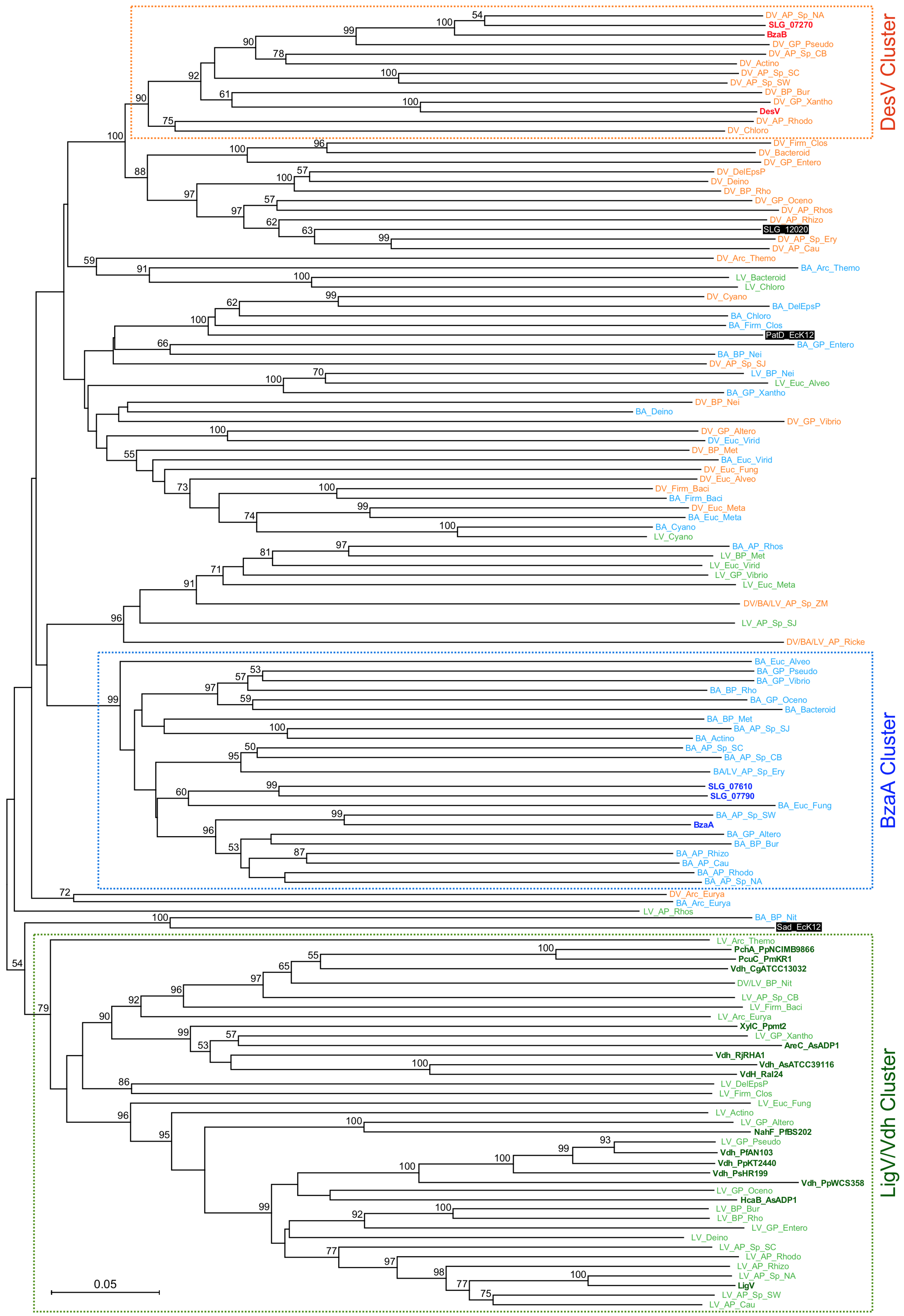

Fig. S10. Phylogenetic tree of DesV, BzaA, LigV, and their related aromatic ALDHs from bacteria, eukaryotes, and archaea. The tree was constructed using the neighbor-joining algorithm

with the Clustal W and MEGA 7 programs employing 1000 bootstrap replicates. Bootstrap values are indicated at the nodes, and the scale corresponds to an evolutionary distance of 0.05 amino acid substitutions per position. The known aromatic ALDHs included in the DesV, BzaA, and LigV/Vdh clusters (Table S4) are shown in red, blue, and green, respectively. ALDHs were selected based on their amino acid sequence similarity with DesV (orange), BzaA (cyan), and LigV (light green) using the STRING database (Table S5). SLG_12020 from SYK-6 (accession No., BAK65877), succinate semialdehyde dehydrogenase from *E. coli* (Sad, AAC74598), and γ-aminobutyraldehyde dehydrogenase from *E. coli* (PatD, AAC74526) shown in white text on a black background (Table S4) were used as outgroups of the DesV, BzaA, and LigV/Vdh clusters, respectively.

References

1. **Ding W, Si M, Zhang W, Zhang Y, Chen C, Zhang L, Lu Z, Chen S, Shen X.** 2015. Functional characterization of a vanillin dehydrogenase in *Corynebacterium glutamicum*. Sci Rep **5:**8044.
2. **Mitsui R, Hirota M, Tsuno T, Tanaka M.** 2010. Purification and characterization of vanillin dehydrogenases from alkaliphile *Micrococcus* sp. TA1 and neutrophile *Burkholderia cepacia* TM1. FEMS Microbiol Lett **303:**41-47.
3. **Gosling A, Zachariou M, Straffon M.** 2008. Purification and characterization of a 4-hydroxybenzaldehyde dehydrogenase cloned from *Acinetobacter baylyi*. Enzyme Microb Technol **43:**417-422.
4. **Sołobodowska S, Giebułtowicz J, Wolinowska R, Wroczyński P.** 2012. Contribution of ALDH1A1 isozyme to detoxification of aldehydes present in food products. Acta Pol Pharm **69:**1380-1383.
5. **Ishige T, Tani A, Sakai Y, Kato N.** 2000. Long-chain aldehyde dehydrogenase that participates in *n*-alkane utilization and wax ester synthesis in *Acinetobacter* sp. strain M-1. Appl Environ Microbiol **66:**3481-3486.
6. **Hidalgo E, Chen YM, Lin EC, Aguilar J.** 1991. Molecular cloning and DNA sequencing of the *Escherichia coli* K-12 *ald* gene encoding aldehyde dehydrogenase. J Bacteriol **173:**6118-6123.
7. **Ho KK, Weiner H.** 2005. Isolation and characterization of an aldehyde dehydrogenase encoded by the *aldB* gene of *Escherichia coli*. J Bacteriol **187:**1067-1073.
8. **Mihasan M, Stefan M, Hritcu L, Artenie V, Brandsch R.** 2013. Evidence of a plasmid-encoded oxidative xylose-catabolic pathway in *Arthrobacter nicotinovorans* pAO1. Res Microbiol **164:**22-30.
9. **Vedadi M, Szittner R, Smillie L, Meighen E.** 1995. Involvement of cysteine 289 in the catalytic activity of an $NADP^+$-specific fatty aldehyde dehydrogenase from *Vibrio harveyi*. Biochemistry **34:**16725-16732.
10. **Imanaka T, Ohta T, Sakoda H, Widhyastuti N, Matsuoka M.** 1993. Cloning, nucleotide-sequence, and efficient expression of the gene coding for thermostable aldehyde dehydrogenase from *Bacillus-Stearothermophilus*, and characterization of the enzyme. J Ferment Bioeng **76:**161-167.
11. **Brouns SJ, Walther J, Snijders AP, van de Werken HJ, Willemen HL, Worm P, de Vos MG, Andersson A, Lundgren M, Mazon HF, van den Heuvel RH, Nilsson P, Salmon L, de Vos WM, Wright PC, Bernander R, van der Oost J.** 2006. Identification of the missing links in prokaryotic pentose oxidation pathways: evidence for enzyme recruitment. J Biol Chem **281:**27378-27388.
12. **Kok M, Oldenhuis R, van der Linden MP, Meulenberg CH, Kingma J, Witholt B.** 1989. The *Pseudomonas oleovorans alkBAC* operon encodes two structurally related rubredoxins and an aldehyde dehydrogenase. J Biol Chem **264:**5442-5451.
13. **Takenaka S, Murakami S, Shinke R, Hatakeyama K, Yukawa H, Aoki K.** 1997. Novel genes encoding 2-aminophenol 1,6-dioxygenase from *Pseudomonas* species AP-3 growing on 2-aminophenol and catalytic properties of the purified enzyme. J Biol Chem **272:**14727-14732.
14. **Watanabe S, Kodaki T, Makino K.** 2006. A novel α-ketoglutaric semialdehyde dehydrogenase: evolutionary insight into an alternative pathway of bacterial L-arabinose metabolism. J Biol Chem **281:**28876-28888.
15. **Jones RM, Collier LS, Neidle EL, Williams PA.** 1999. *areABC* genes determine the catabolism of aryl esters in *Acinetobacter* sp. strain ADP1. J Bacteriol **181:**4568-4575.
16. **Itoh Y.** 1997. Cloning and characterization of the *aru* genes encoding enzymes of the catabolic arginine succinyltransferase pathway in *Pseudomonas aeruginosa*. J Bacteriol **179:**7280-7290.
17. **Schneider BL, Kiupakis AK, Reitzer LJ.** 1998. Arginine catabolism and the arginine succinyltransferase pathway in

Escherichia coli. J Bacteriol **180:**4278-4286.

18. **Yao X, He W, Lu CD.** 2011. Functional characterization of seven γ-glutamylpolyamine synthetase genes and the *bauRABCD* locus for polyamine and β-alanine utilization in *Pseudomonas aeruginosa* PAO1. J Bacteriol **193:**3923-3930.
19. **Boyd LA, Adam L, Pelcher LE, McHughen A, Hirji R, Selvaraj G.** 1991. Characterization of an *Escherichia coli* gene encoding betaine aldehyde dehydrogenase (BADH): structural similarity to mammalian ALDHs and a plant BADH. Gene **103:**45-52.
20. **Pocard JA, Vincent N, Boncompagni E, Smith LT, Poggi MC, Le Rudulier D.** 1997. Molecular characterization of the *bet* genes encoding glycine betaine synthesis in *Sinorhizobium meliloti* 102F34. Microbiology **143 (Pt 4):**1369-1379.
21. **Gescher J, Ismail W, Olgeschläger E, Eisenreich W, Wörth J, Fuchs G.** 2006. Aerobic benzoyl-coenzyme A (CoA) catabolic pathway in *Azoarcus evansii*: conversion of ring cleavage product by 3,4-dehydroadipyl-CoA semialdehyde dehydrogenase. J Bacteriol **188:**2919-2927.
22. **Achterholt S, Priefert H, Steinbüchel A.** 1998. Purification and characterization of the coniferyl aldehyde dehydrogenase from *Pseudomonas* sp. strain HR199 and molecular characterization of the gene. J Bacteriol **180:**4387-4391.
23. **Díaz-Sánchez V, Estrada AF, Trautmann D, Al-Babili S, Avalos J.** 2011. The gene *carD* encodes the aldehyde dehydrogenase responsible for neurosporaxanthin biosynthesis in *Fusarium fujikuroi*. FEBS J **278:**3164-3176.
24. **Iwaki H, Hasegawa Y, Teraoka M, Tokuyama T, Bergeron H, Lau PC.** 1999. Identification of a transcriptional activator (ChnR) and a 6-oxohexanoate dehydrogenase (ChnE) in the cyclohexanol catabolic pathway in *Acinetobacter* sp. strain NCIMB 9871 and localization of the genes that encode them. Appl Environ Microbiol **65:**5158-5162.
25. **Brzostowicz PC, Walters DM, Thomas SM, Nagarajan V, Rouvière PE.** 2003. mRNA differential display in a microbial enrichment culture: simultaneous identification of three cyclohexanone monooxygenases from three species. Appl Environ Microbiol **69:**334-342.
26. **Eaton RW.** 1997. *p*-Cymene catabolic pathway in *Pseudomonas putida* F1: cloning and characterization of DNA encoding conversion of *p*-cymene to *p*-cumate. J Bacteriol **179:**3171-3180.
27. **Su M, Li Y, Ge X, Tian P.** 2015. 3-Hydroxypropionaldehyde-specific aldehyde dehydrogenase from *Bacillus subtilis* catalyzes 3-hydroxypropionic acid production in *Klebsiella pneumoniae*. Biotechnol Lett **37:**717-724.
28. **Schwibbert K, Marin-Sanguino A, Bagyan I, Heidrich G, Lentzen G, Seitz H, Rampp M, Schuster SC, Klenk HP, Pfeiffer F, Oesterhelt D, Kunte HJ.** 2011. A blueprint of ectoine metabolism from the genome of the industrial producer *Halomonas elongata* DSM 2581^T. Environ Microbiol **13:**1973-1994.
29. **Ferrández A, Prieto MA, García JL, Díaz E.** 1997. Molecular characterization of PadA, a phenylacetaldehyde dehydrogenase from *Escherichia coli*. FEBS Lett **406:**23-27.
30. **Donnelly MI, Cooper RA.** 1981. Succinic semialdehyde dehydrogenases of *Escherichia coli*: their role in the degradation of *p*-hydroxyphenylacetate and γ-aminobutyrate. Eur J Biochem **113:**555-561.
31. **Espinosa-Urgel M, Ramos JL.** 2001. Expression of a *Pseudomonas putida* aminotransferase involved in lysine catabolism is induced in the rhizosphere. Appl Environ Microbiol **67:**5219-5224.
32. **Jang EH, Park SA, Chi YM, Lee KS.** 2014. Kinetic and structural characterization for cofactor preference of succinic semialdehyde dehydrogenase from *Streptococcus pyogenes*. Mol Cells **37:**719-726.
33. **Asanuma N, Hino T.** 2006. Presence of NAD^+-specific glyceraldehyde-3-phosphate dehydrogenase and CcpA-dependent transcription of its gene in the ruminal bacterium *Streptococcus bovis*. FEMS Microbiol Lett **257:**17-23.
34. **Brunner NA, Brinkmann H, Siebers B, Hensel R.** 1998. NAD^+-dependent glyceraldehyde-3-phosphate dehydrogenase from

Thermoproteus tenax. The first identified archaeal member of the aldehyde dehydrogenase superfamily is a glycolytic enzyme with unusual regulatory properties. J Biol Chem **273:**6149-6156.

35. **Boch J, Kempf B, Schmid R, Bremer E.** 1996. Synthesis of the osmoprotectant glycine betaine in *Bacillus subtilis*: characterization of the *gbsAB* genes. J Bacteriol **178:**5121-5129.
36. **Prieto MA, Díaz E, García JL.** 1996. Molecular characterization of the 4-hydroxyphenylacetate catabolic pathway of *Escherichia coli* W: engineering a mobile aromatic degradative cluster. J Bacteriol **178:**111-120.
37. **Roper DI, Stringfellow JM, Cooper RA.** 1995. Sequence of the *hpcC* and *hpcG* genes of the *meta*-fission homoprotocatechuic acid pathway of *Escherichia coli* C: nearly 40% amino-acid identity with the analogous enzymes of the catechol pathway. Gene **156:**47-51.
38. **Stines-Chaumeil C, Talfournier F, Branlant G.** 2006. Mechanistic characterization of the MSDH (methylmalonate semialdehyde dehydrogenase) from *Bacillus subtilis*. Biochem J **395:**107-115.
39. **Aghaie A, Lechaplais C, Sirven P, Tricot S, Besnard-Gonnet M, Muselet D, de Berardinis V, Kreimeyer A, Gyapay G, Salanoubat M, Perret A.** 2008. New insights into the alternative D-glucarate degradation pathway. J Biol Chem **283:**15638-15646.
40. **Johnsen U, Dambeck M, Zaiss H, Fuhrer T, Soppa J, Sauer U, Schonheit P.** 2009. D-xylose degradation pathway in the halophilic archaeon *Haloferax volcanii*. J Biol Chem **284:**27290-27303.
41. **McLeish MJ, Kneen MM, Gopalakrishna KN, Koo CW, Babbitt PC, Gerlt JA, Kenyon GL.** 2003. Identification and characterization of a mandelamide hydrolase and an $NAD(P)^+$-dependent benzaldehyde dehydrogenase from *Pseudomonas putida* ATCC 12633. J Bacteriol **185:**2451-2456.
42. **Steele MI, Lorenz D, Hatter K, Park A, Sokatch JR.** 1992. Characterization of the *mmsAB* operon of *Pseudomonas aeruginosa* PAO encoding methylmalonate-semialdehyde dehydrogenase and 3-hydroxyisobutyrate dehydrogenase. J Biol Chem **267:**13585-13592.
43. **Heinaru E, Truu J, Stottmeister U, Heinaru A.** 2000. Three types of phenol and *p*-cresol catabolism in phenol- and *p*-cresol-degrading bacteria isolated from river water continuously polluted with phenolic compounds. FEMS Microbiol Ecol **31:**195-205.
44. **Samsonova NN, Smirnov SV, Novikova AE, Ptitsyn LR.** 2005. Identification of *Escherichia coli* K12 YdcW protein as a γ-aminobutyraldehyde dehydrogenase. FEBS Lett **579:**4107-4112.
45. **Cronin CN, Kim J, Fuller JH, Zhang X, McIntire WS.** 1999. Organization and sequences of *p*-hydroxybenzaldehyde dehydrogenase and other plasmid-encoded genes for early enzymes of the *p*-cresol degradative pathway in *Pseudomonas putida* NCIMB 9866 and 9869. DNA Seq **10:**7-17.
46. **Wright A, Olsen RH.** 1994. Self-mobilization and organization of the genes encoding the toluene metabolic pathway of *Pseudomonas mendocina* KR1. Appl Environ Microbiol **60:**235-242.
47. **Arias S, Olivera ER, Arcos M, Naharro G, Luengo JM.** 2008. Genetic analyses and molecular characterization of the pathways involved in the conversion of 2-phenylethylamine and 2-phenylethanol into phenylacetic acid in *Pseudomonas putida* U. Environ Microbiol **10:**413-432.
48. **Iwabuchi T, Harayama S.** 1997. Biochemical and genetic characterization of 2-carboxybenzaldehyde dehydrogenase, an enzyme involved in phenanthrene degradation by *Nocardioides* sp. strain KP7. J Bacteriol **179:**6488-6494.
49. **Kiyohara H, Nagao K, Yano K.** 1981. Isolation and some properties of NAD-linked 2-carboxybenzaldehyde dehydrogenase in *Alcaligenes faecalis* AFK 2 grown on phenanthrene. J Gen Appl Microbiol **27:**443-455.

50. **Simon O, Klaiber I, Huber A, Pfannstiel J.** 2014. Comprehensive proteome analysis of the response of *Pseudomonas putida* KT2440 to the flavor compound vanillin. J Proteomics **109:**212-227.

51. **Jo JE, Mohan Raj S, Rathnasingh C, Selvakumar E, Jung WC, Park S.** 2008. Cloning, expression, and characterization of an aldehyde dehydrogenase from *Escherichia coli* K-12 that utilizes 3-hydroxypropionaldehyde as a substrate. Appl Microbiol Biotechnol **81:**51-60.

52. **Luo M, Singh RK, Tanner JJ.** 2013. Structural determinants of oligomerization of Δ^1-pyrroline-5-carboxylate dehydrogenase: identification of a hexamerization hot spot. J Mol Biol **425:**3106-3120.

53. **Donnelly MI, Cooper RA.** 1981. Two succinic semialdehyde dehydrogenases are induced when *Escherichia coli* K-12 is grown on γ-aminobutyrate. J Bacteriol **145:**1425-1427.

54. **Krejcik Z, Denger K, Weinitschke S, Hollemeyer K, Paces V, Cook AM, Smits TH.** 2008. Sulfoacetate released during the assimilation of taurine-nitrogen by *Neptuniibacter caesariensis*: purification of sulfoacetaldehyde dehydrogenase. Arch Microbiol **190:**159-168.

55. **Beltrametti F, Marconi AM, Bestetti G, Colombo C, Galli E, Ruzzi M, Zennaro E.** 1997. Sequencing and functional analysis of styrene catabolism genes from *Pseudomonas fluorescens* ST. Appl Environ Microbiol **63:**2232-2239.

56. **Junker F, Kiewitz R, Cook AM.** 1997. Characterization of the *p*-toluenesulfonate operon *tsaMBCD* and *tsaR* in *Comamonas testosteroni* T-2. J Bacteriol **179:**919-927.

57. **Arcos M, Olivera ER, Arias S, Naharro G, Luengo JM.** 2010. The 3,4-dihydroxyphenylacetic acid catabolon, a catabolic unit for degradation of biogenic amines tyramine and dopamine in *Pseudomonas putida* U. Environ Microbiol **12:**1684-1704.

58. **Fleige C, Hansen G, Kroll J, Steinbüchel A.** 2013. Investigation of the *Amycolatopsis* sp. strain ATCC 39116 vanillin dehydrogenase and its impact on the biotechnical production of vanillin. Appl Environ Microbiol **79:**81-90.

59. **Beltrametti F, Marconi AM, Bestetti G, Colombo C, Galli E, Ruzzi M, Zennaro E.** 1997. Sequencing and functional analysis of styrene catabolism genes from *Pseudomonas fluorescens* ST. Appl Environ Microbiol **63:**2232-2239.

60. **Plaggenborg R, Overhage J, Steinbüchel A, Priefert H.** 2003. Functional analyses of genes involved in the metabolism of ferulic acid in *Pseudomonas putida* KT2440. Appl Microbiol Biotechnol **61:**528-535.

61. **Venturi V, Zennaro F, Degrassi G, Okeke BC, Bruschi CV.** 1998. Genetics of ferulic acid bioconversion to protocatechuic acid in plant-growth-promoting *Pseudomonas putid*a WCS358. Microbiology **144 (Pt 4):**965-973.

62. **Priefert H, Rabenhorst J, Steinbüchel A.** 1997. Molecular characterization of genes of *Pseudomonas* sp. strain HR199 involved in bioconversion of vanillin to protocatechuate. J Bacteriol **179:**2595-2607.

63. **Plaggenborg R, Overhage J, Loos A, Archer JA, Lessard P, Sinskey AJ, Steinbüchel A, Priefert H.** 2006. Potential of *Rhodococcus* strains for biotechnological vanillin production from ferulic acid and eugenol. Appl Microbiol Biotechnol **72:**745-755.

64. **Chen HP, Chow M, Liu CC, Lau A, Liu J, Eltis LD.** 2012. Vanillin catabolism in *Rhodococcus jostii* RHA1. Appl Environ Microbiol **78:**586-588.

65. **Inoue J, Shaw JP, Rekik M, Harayama S.** 1995. Overlapping substrate specificities of benzaldehyde dehydrogenase (the *xylC* gene product) and 2-hydroxymuconic semialdehyde dehydrogenase (the *xylG* gene product) encoded by TOL plasmid pWW0 of *Pseudomonas putida*. J Bacteriol **177:**1196-1201.

66. **Katayama Y, Nishikawa S, Nakamura M, Yano K, Yamasaki M, Morohoshi N, Haraguchi T.** 1987. Cloning and expression of *Pseudomonas paucimobilis* SYK-6 genes involved in the degradation of vanillate and protocatechuate in *Pseudomonas putida*. Mokuzai Gakkaishi **33:**77-79.

67. **Masai E, Yamamoto Y, Inoue T, Takamura K, Hara H, Kasai D, Katayama Y, Fukuda M.** 2007. Characterization of *ligV* essential for catabolism of vanillin by *Sphingomonas paucimobilis* SYK-6. Biosci Biotechnol Biochem **71:**2487-2492.

68. **Yanisch-Perron C, Vieira J, Messing J.** 1985. Improved M13 phage cloning vectors and host strains: nucleotide sequences of the M13mp18 and pUC19 vectors. Gene **33:**103-119.

69. **Studier FW, Moffatt BA.** 1986. Use of bacteriophage T7 RNA polymerase to direct selective high-level expression of cloned genes. J Mol Biol **189:**113-130.

70. **Short JM, Fernandez JM, Sorge JA, Huse WD.** 1988. λ ZAP: a bacteriophage λ expression vector with *in vivo* excision properties. Nucleic Acids Res **16:**7583-7600.

71. **Schäfer A, Tauch A, Jäger W, Kalinowski J, Thierbach G, Pühler A.** 1994. Small mobilizable multi-purpose cloning vectors derived from the *Escherichia coli* plasmids pK18 and pK19: selection of defined deletions in the chromosome of *Corynebacterium glutamicum*. Gene **145:**69-73.

72. **Masai E, Sasaki M, Minakawa Y, Abe T, Sonoki T, Miyauchi K, Katayama Y, Fukuda M.** 2004. A novel tetrahydrofolate-dependent *O*-demethylase gene is essential for growth of *Sphingomonas paucimobilis* SYK-6 with syringate. J Bacteriol **186:**2757-2765.

73. **Blatny JM, Brautaset T, Winther-Larsen HC, Karunakaran P, Valla S.** 1997. Improved broad-host-range RK2 vectors useful for high and low regulated gene expression levels in gram-negative bacteria. Plasmid **38:**35-51.

74. **Fukuhara Y, Kamimura N, Nakajima M, Hishiyama S, Hara H, Kasai D, Tsuji Y, Narita-Yamada S, Nakamura S, Katano Y, Fujita N, Katayama Y, Fukuda M, Kajita S, Masai E.** 2013. Discovery of pinoresinol reductase genes in sphingomonads. Enzyme Microb Technol **52:**38-43.
